# System immunoinformatics–based design of a multi-epitope vaccine candidate against La Crosse virus

**DOI:** 10.1371/journal.pone.0350287

**Published:** 2026-05-28

**Authors:** Md. Habib Ullah Masum, Homaira Pervin Heema, Ahmad Abdullah Mahdeen, Syed Mohammad Lokman, Zarin Tasnim, Md. Rakibul Hasan, Sanjida Hossain Arpa, Rehana Parvin, Jannatul Ferdous, Mohammad Sharif Uddin

**Affiliations:** 1 Department of Genomics and Bioinformatics, Faculty of Biotechnology and Genetic Engineering, Chattogram Veterinary and Animal Sciences University, Chattogram, Bangladesh; 2 Department of Pathology and Parasitology, Faculty of Veterinary Medicine, Chattogram Veterinary and Animal Sciences University, Chattogram, Bangladesh; 3 Department of Microbiology, Notre Dame University Bangladesh (NDUB), Dhaka, Bangladesh; 4 Asian University for Women, Chattogram, Bangladesh; 5 Department of Industrial Biotechnology, Faculty of Biotechnology and Genetic Engineering, Chattogram Veterinary and Animal Sciences University, Chattogram, Bangladesh; 6 Department of Microbiology, Noakhali Science and Technology University, Noakhali, Bangladesh; 7 Department of Obstetrics and Gynecology, Chittagong Medical College Hospital, Chattogram, Bangladesh‌‌; Bangladesh Council of Scientific and Industrial Research, BANGLADESH

## Abstract

The emergence of La Crosse virus (LACV) infection has been attributed to the global expansion of mosquito habitats and climate change. This pressing issue underscores the urgency of preemptive actions to combat the infection. Currently, no vaccines or targeted antivirals are available for LACV infection. The present study utilizes advanced immunoinformatics approaches for the design of a multiepitope vaccine (LACV-mVax01) targeting the viral proteins G1, G2, and N. The structural assessment suggested favorable stability characteristics, reflected by an instability score of 17.12 and an aliphatic index of 81.88. Based on the two-dimensional structure, the LACV-mVax01 had an alpha-helical content of ~45%, with favorable beta sheets and coils. The refinement of the LACV-mVax01 resulted in an accurate model (TM-score 0.49, C-score −1.86), supported by high-quality metrics (RMSD 0.489, MolProbity 2.230, ERRAT 85.539). Docking analyses indicated potential binding interactions with TLR4, showing a binding energy of −1000.0 compared to −939.2 for TLR2. Molecular dynamics simulations were consistent with these observations, suggesting relatively stable interactions and maintenance of structural integrity in both complexes. Codon optimization yielded a codon adaptation index of 0.9848, and the predicted mRNA structure had a free energy of −342.16 kcal/mol, consistent with a stable conformation. Subsequent *in silico* immunological analyses suggested humoral and cellular responses, including probable memory B- and T-cell responses. However, sustained immunity was also supported by consecutive cytokine and antibody synthesis. The comprehensive structural, molecular, and immunological evaluations suggest that LACV-mVax01 is a promising vaccine candidate capable of eliciting sustained protective immunity against LACV infection. Though future *in vitro*, *in vivo*, and clinical validations are required to perceive its effectiveness and safety.

## 1. Introduction

La Crosse virus (LACV) is an RNA virus classified in the California serogroup of the *Orthobunyavirus* genus [1]. This virus came into the limelight in the 1960s with the potential to cause severe human diseases [2,3]. However, the virus most frequently causes pediatric arboviral encephalitis in the United States (US). It is transmitted to humans by the bite of infected mosquitoes, with the definitive vector being the *Aedes triseriatus* mosquito, often encountered in the eastern region [4]. Eastern chipmunks (*Tamias striatus grinseus*) and Eastern gray squirrels (*Sciurus carolinensis*) serve as amplifying hosts for LACV while providing a food source for the mosquitoes. Notably, the virus may persist in the mosquito without a vertebrate host via transovarial transmission, enabling it to endure the winter in mosquito eggs [5]. The incidence of encephalitis in the US is increasing rapidly due to the emergence of LACV [6]. It is estimated that over 300,000 infections arise annually, yet only a few of these cases are deemed severe [6]. In its severe manifestation, LACV infections lead to LACV neuroinvasive disease (LACV-ND), characterized by meningoencephalitis, which often results in enduring neurological complications, such as reduced cognitive function and chronic seizure disorders, with an anticipated mortality rate between 0.5% and 1.9%. The significant impact of LACV is exacerbated by the lack of effective treatments, such as effective vaccines or antivirals [7].

The LACV virions possess pleiomorphic morphology with a diameter of approximately 100 nm. The virus has a negative-sense segmented RNA genome consisting of three RNA segments: short (S), medium (M), and large (L) [8,9]. The L segment comprises an open reading frame (ORF) for the RNA-dependent RNA polymerase (RdRp), which initiates its mRNA synthesis using host-cell 5’ mRNA sequences. This process leads to the halt of host cell protein synthesis following LACV infection [10]. Besides, the M segment contains only ORF, which is transformed into surface transmembrane Glycoprotein 1 (G1), Glycoprotein 2 (G2), and a non-structural protein (NSm) [11]. Among them, G1 and G2 proteins promote LACV entry into the host cell via clathrin-mediated endocytosis, during which they engage host cell surface receptors [12]. After the viral entry, the LACV is transported into early endosomes by Rab-5 [13], and incorporated into the Golgi complex, which facilitates further viral assembly [14]. The S segment encodes two proteins: nucleoprotein (N) and non-structural protein (NSs), which antagonize the mammalian type I interferon system. The N protein and the viral RdRp facilitate viral replication and transcription activities. It has also been associated with modulating host immune responses by disrupting interferon (IFN) signalling pathways and promoting viral persistence [15,16]. Despite the significant threat to human health, no antiviral treatments or vaccines are available to treat the infection [17]. Vaccination against LACV has been explored in animal research; however, human clinical trials have not yet been conducted [18].

In this study, a multiepitope vaccine against LACV was designed using immunoinformatics approaches. The vaccine design includes several epitopes obtained from the G1, G2, and N proteins of LACV, which are conjugated with an adjuvant to augment immune responses. Hence, this vaccine could be a viable vaccine option for LACV infections.

## 2. Materials and methods

### 2.1. Retrieval of pathogenic proteins and phylogenetic tree construction

After a comprehensive literature review, the G1, G2, and N of the LACV were selected for the vaccine design. The amino acid sequences of the M polyprotein (accession: YP_010839410.1) and nucleoprotein N (accession: YP_010839408.1) were retrieved in FASTA format from the NCBI protein database. Regarding the M polyprotein, the amino acid sequences from 510–1380 and 19–299 were selected as the representative regions of the G1 and G2 proteins, respectively. Afterwards, protein-protein BLAST was executed on NCBI for every targeted protein. From the BLAST result, 36 hits of G1 protein sequences, 37 hits of G2 protein sequences, and 15 hits of N protein sequences were obtained from various LACV strains globally and stored in FASTA format [19]. Next, the consensus sequences were generated from all the selected protein (G1, G2, and N) sequences using the BioEdit 7.2 program [20]. This could lead to developing a very effective vaccine among the strains. The phylogenetic analysis between the strains of LACV was performed for all the proteins sequences using the MEGA 11 software [21]. The consensus sequences of the respective G1, G2, and N proteins were applied for the subsequent analysis and stored as separate FASTA files.

### 2.2. Major histocompatibility complex-I (MHC-I), and major histocompatibility complex-II (MHC-II) epitope prediction

The binding epitopes of selected LACV proteins—G1, G2, and N proteins—were systematically analyzed for both MHC-I and MHC-II epitope prediction. The MHC-I binding epitopes, along with proteosome, TAP transport, and processing scores, were predicted using the Immune Epitope Database (IEDB) and the NetMHC 4.0 server [22,23]. For MHC-II binding epitopes, a robust prediction system was implemented using the IEDB and the NetMHCIIpan 4.0 server [22,23]. We further utilized the VaxiJen 2.0 server to determine the antigenic features of the chosen epitopes [24], while the AllerTOP v.2.0 [25] and ToxinPred server (https://webs.iiitd.edu.in/raghava/toxinpred/algo.php) were used to identify potential allergenic and toxic effects [26]. The interferon-gamma (IFN-g) induction of the chosen epitopes was also predicted using the IFNepitope server. Epitopes that met all of the requirements were then chosen for the vaccine development process [27].

### 2.3. Linear B-lymphocyte (LBL) epitope prediction

The IEDB (http://tools.iedb.org/bcell/) and ABCpred (http://crdd.osdd.net/raghava/abcpred/) servers were implemented to accurately forecast the LBL epitopes of the targeted proteins (G1, G2, and N proteins). Finally, the VaxiJen 2.0, AllerTOP v. 2.0 [25] and ToxinPred server [26] underwent to screen the antigenic potentiality, allergenic and toxic effects of the selected epitopes.

### 2.4. Population coverage analysis

The prevalence of HLA alleles differs based on variables such as ethnicity and geographic region [28,29]. The IEDB population coverage resource provides the global population coverage of epitopes (http://tools.iedb.org/population/) [30]. In this process the appropriate MHC alleles alongside the chosen MHC-I and MHC-II epitopes was utilized from the targeted proteins.

### 2.5. Mapping and biophysical features of the LACV-mVax01 vaccine

The mapping of the LACV-mVax01 was performed using the adjuvant Large ribosomal subunit protein bL12 Mycobacterium tuberculosis (sp|P9WHE3) with the highly prioritized epitopes of G1, G2, and N proteins. Moreover, EAAAK, AYY, AK, and KFER were the four linkers that interlinked the synthesized vaccine. We have designated the vaccine as LACV-mVax01, where LACV denotes the virus, mVax indicates vaccine, and 01 refers to its first version. The biophysical characteristics of LACV-mVax01 were analyzed using the Expasy ProtParam server. This server provides various pieces of information, including the grand average of hydropathicity (GRAVY), isoelectric point (pI), instability and aliphatic index, instability index, total amino acid count, molecular weight, and total atom count [31]. SOLpro and SOSUI were subsequently identified as the most effective servers to predict the solubility of the vaccine [32–34]. However, the allergenic feature of the LACV-mVax01 was evaluated using AllergenFP v.1.0 [35], AllerTOP v. 2.0 [25] and AlgPred [36] server. While the antigenicity prediction of the LACV-mVax01 was conducted by ANTIGENpro [27], and VaxiJen 2.0 server [24]. At the end, the toxicity of the LACV-mVax01 was assessed by the ToxinPred server [26].

### 2.6. Structural prediction and validation

The two-dimensional structure of the LACV-mVax01 was predicted using PSIPRED (http://bioinf.cs.ucl.ac.uk/psipred/) server [37], where the server employs position-specific prediction-based (PSI–BLAST) approach. Furthermore, the SOPMA and the GOR4 were applied to evaluate the two-dimensional structure of the LACV-mVax01 [6,38–40]. Nonetheless, the SOPMA server can identify around 69.5% of amino acids based on the protein sequence and the three-mode representation (alpha-helix, beta-sheet, and coil structures) [40]. However, the GOR4 server employs Bayesian statistics and information theory to approximate the two-dimensional structure of proteins [41]. The I-TASSER server, which uses iterative simulations of template fragment assembly and alternative threading alignments, was assigned with the three-dimensional prediction of the LACV-mVax01 structure (https://zhanggroup.org/I-TASSER/) [42,43]. The GalaxyWEB server was employed to further refinement of the predicted model of the LACV-mVax01 [44]. Furthermore, the SAVES v6.0 server was utilized to conduct structural validation of the refined LACV-mVax01 model [45–48]. To figure out any ambiguities of the predicted LACV-mVax01 model, we utilized the ProSA-web server, where the Z-score indicates the accuracy of the predicted model [49,50].

### 2.7. Prediction of conformational B-lymphocyte (CBL) epitopes

When it comes to the CBL epitope prediction of the LACV-mVax01, both the DiscoTope 2.0 [51] and the Ellipro servers [52] were applied. During the prediction, the ElliPro utilizes a combination of three different approaches [52], whereas the DiscoTope 2.0 server depends on surface accessibility measures [51].

### 2.8. Molecular docking analysis of the LACV-mVax01 with the TLRs

The molecular docking study was conducted by the CLUSPRO 2.0 server [53–56]. Before starting this analysis, the three-dimensional structures of human TLR2 (Toll-like receptor 2) (PDB: 2Z7X) and human TLR4 (Toll-like receptor 4) (PDB: 3FXI) were retrieved from the Protein Data Bank (PDB) database (www.rcsb.org). The TLR-2 and TLR-4 are play key roles in vaccine-mediated immunity by detecting pathogen-associated molecular patterns (PAMPs) and activating both innate and adaptive immune responses [57]. TLR-2 primarily recognizes lipoproteins and lipopeptides, while TLR-4 identifies lipopolysaccharides (LPS). Activation of TLR-2 and TLR-4 by vaccine antigens triggers downstream signaling pathways that enhance adaptive immunity [58]. This activation increases antigen presentation, cytokine production, and dendritic cell maturation, which collectively promote antibody production, T-cell activation, and the establishment of immunological memory, thereby increasing vaccine efficacy [57,58]. Subsequently, the docked complexes were assessed and visualized using PyMOL (https://pymol.org/2) and PDBsum (http://www.ebi.ac.uk/thornton-srv/databases/pdbsum/Generate.html).

### 2.9. Molecular dynamics simulation and post-simulation analysis

The molecular dynamics simulation analysis of the LACV-mVax01_apo, LACV-mVax01_TLR2, and LACV-mVax01_TLR4 was carried out by the GROMACS (version 2022.3) program [59]. For this simulation, a water box was built with the OPC water model [60,61] in conjunction with the GROMOS96 43a1 force field [62]. The systems underwent equilibration, starting with constant volume and temperature (NVT) conditions at 300 K, using a modified Berendsen thermostat [63]. This was followed by equilibration under constant volume and pressure (NPT) conditions at 1 atm, facilitated by a Berendsen barostat [64]. Both NVT and NPT equilibrations were conducted for 1 ns each. After equilibration, the systems proceeded to a 100 ns production phase of molecular dynamics simulations. During this production phase, the temperature was maintained at 300 K using a modified Berendsen thermostat [65], while pressure was held at 1 atm with the Parrinello-Rahman barostat. Covalent bonds were constrained using the LINCS algorithm [66]. Long-range electrostatic interactions were calculated using the Particle Mesh Ewald (PME) method with a cut-off of 1.2 nm. After the simulations, periodic boundary conditions (PBC) were removed from the output trajectories, which were subsequently utilized for analysis [67]. The integrated modules of the GROMACS program were responsible for performing calculations such as the root mean square deviation (RMSD), radius of gyration (ROG), solvent accessible surface area (SASA), and hydrogen bond (H-bond) analysis. The ggplot2 function in RStudio was employed to visualize the outcomes of each analysis. The visual molecular dynamics (VMD) [68] and MMPBSA.py [69] package of AMBER 18 program was utilized to execute the MMPBSA for the LACV-mVax01_apo, LACV-mVax01_TLR2, and LACV-mVax01_TLR4 complexes [69]. This molecular mechanic approach, like MMGBSA, evaluates several intermolecular interactions, including total binding affinities (ΔTOTAL), electrostatic interactions (ΔEEL), van der Waals forces (ΔVDWAALS), polar (ΔEGB), and non-polar (ΔESURF) components [70–72].

### 2.10. Optimization of codon and virtual molecular cloning

Utilizing the *E. coli* K12 strain, the Java Codon Adaptation program (JCat) was applied to improve the LACV-mVax01’s coding sequences (http://www.jcat.de/Start.jsp). The server predicts the expression level of a protein using the adaptation index (CAI) and the GC content, where the acceptable CAI score is ≥ 0.8 and GC may vary from 30% to 70%. There may be a transcriptional and translational efficacy reduction once these thresholds are surpassed [73,74]. The restriction sites Eco53kI and EcoRV were added into the N-terminal and C-terminal regions of the LACV-mVax01 sequence, respectively, following the cloning of the optimized vaccine gene sequence into the *E. coli* plasmid vector pET-28a(+). The optimized sequence of the LACV-mVax01 was subsequently stipulated into the plasmid vector pET-28a(+), which was subjected to virtual molecular cloning using the SnapGene software.

### 2.11. Immune simulation and in silico cloning

The C-ImmSim server is an optimized server to perform *in silico* immunological simulations of any vaccine construct [75]. This server elucidates the humoral responses mediated by antibodies alongside the cellular responses mediated by cells within the mammalian immune system in the context of vaccination [74,76]. Before the immunization was given, the LACV-mVax01 had been designed to include three doses administered at four-week intervals. The simulation parameters remained at their default values, with the number of adjuvants and antigen injections configured to 100 and 1000, respectively. The time steps were also established at 1, 84, and 168. The default configuration utilized the random seed (12,345) without including lipopolysaccharides (LPS), while the simulation volume and steps were set to 50 and 1000, respectively.

### 2.12. mRNA structure prediction

To predict the two-dimensional structures of the LACV-mVax01, the RNAfold server was employed [77]. The server estimates the minimum free energy (MFE) of the searching mRNA structures through thermodynamic calculations [78,79]. The LACV-mVax01 DNA sequences were optimized using JCat and transcribed to RNA with the DNA- > RNA- > Protein conversion approach (http://biomodel.uah.es/en/lab/cybertory/analysis/trans.htm). Subsequently, the RNA sequence was used in the RNAfold server for two-dimensional structure prediction and validation.

## 3. Results

### 3.1. Retrieval of pathogenic proteins and phylogenetic tree construction

**Fig 1:**
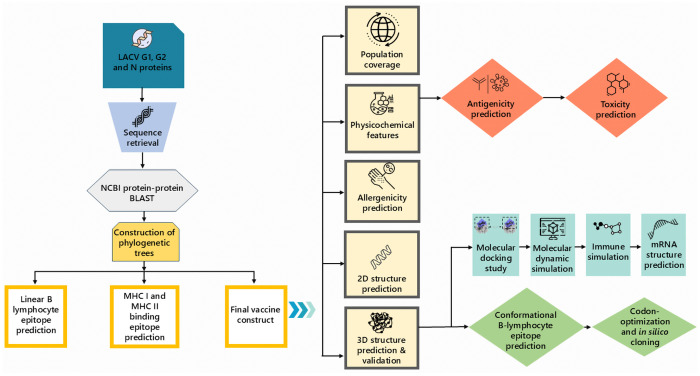
The roadmap of the novel multiepitope vaccine design against LACV.

The NCBI database was accessed to retrieve the reference sequences of the LACV G1, G2, and N. After the protein-protein BLAST, the consensus sequences of each of the proteins (G1, G2, and N) were used for the subsequent analysis in this study (S1 and S2 Figs). The research roadmap for designing a multiepitope vaccine against LACV (LACV-mVax01) is depicted in Fig 1. The phylogenetic tree was constructed using the Neighbour-Joining (NJ) algorithm (S3 Fig).

### 3.2. MHC-I and MHC-II epitope prediction

A total of six nine-mer and a total of six fifteen-mer length peptides of MHC-I and MHC-II were selected based on their percentile rank (≤1.00) and affinity score, respectively (S1 and S2 Tables). Furthermore, the selected peptide sequences satisfied the requirements for being antigenic, non-allergic, and non-toxic. Additionally, the selected peptide sequences were secure and did not induce any toxicity or allergic reactions. The predicted proteasomal cleavage, TAP transport efficiency, and overall antigen processing scores were found to be favorable for the selected MHC-1 epitopes (S1 Table). Moreover, it was observed that the MHC-II epitopes could induce IFN-γ, with no indication of IL-10 induction (S2 Table).

### 3.3. LBL epitope prediction

A total of six peptide sequences from the G1, G2, and N proteins were selected to serve as linear epitopes for B-lymphocytes. Antigenic, non-allergenic, and non-toxic peptide sequences were selected for the final vaccine construct (S3 Table).

### 3.4. Population coverage analysis

The IEDB server was used to evaluate the worldwide population coverage of certain MHC-I and MHC-II epitopes. The findings indicated that approximately 98.55% and 99.99% of the world population may be covered by MHC-I and MHC-II epitopes, respectively. Remarkably, the analysis demonstrated complete (100%) coverage of the population when both MHC-I and MHC-II epitopes were combined. The regional analysis highlighted the extensive reach of these epitopes, with complete population coverage (100%) observed in East Asia, Northeast Asia, South Asia, Europe, East Africa, West Africa, Central Africa, North Africa, the West Indies, North America, South America, Central America, and Oceania. Near-complete coverage was also recorded in Southwest Asia (99.99%) and Southeast Asia (99.98%). The only notable exception was South Africa, where the coverage was slightly lower at 95.27%.

### 3.5. Mapping and biophysical features of the LACV-mVax01 vaccine

**Fig 2:**
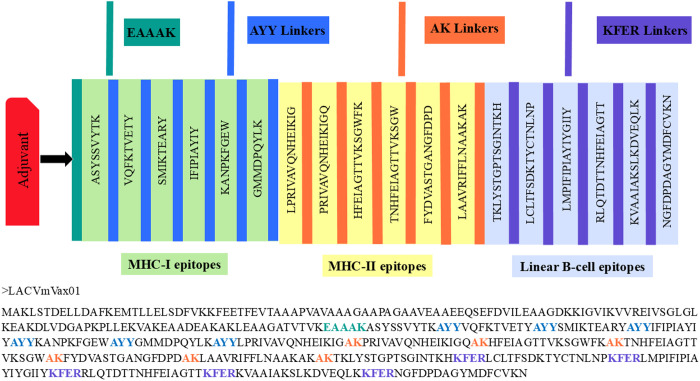
The LACV-mVax01 construct. The figure depicts a peptide sequence of 425 amino acids, with adjuvants (red), MHC-I epitopes (light green), MHC-II (yellow), LBL (light blue) epitopes, and linkers (EAAAK in gray, AYY in dark blue, AK in orange, and KFER in purple).

The vaccine formulation included the adjuvant large ribosomal subunit protein bL12 from Mycobacterium tuberculosis (sp|P9WHE3), with key epitopes derived from the G1, G2, and N proteins. Furthermore, four linkers—EAAAK, AYY, AK, and KFER—were used to interconnect the synthesized vaccine components. The vaccine is designated LACV-mVax01, with LACV indicating the virus, “mVax” denoting immunization, and “01” representing the first version (Fig 2). Utilizing the ExPASy ProtPram database, the physicochemical properties of the LACV-mVax01 were examined. The server reports that the LACV-mVax01 has 425 amino acids and a molecular weight of 47189.42 Da. The LACV-mVax01 has a pI of 8.99, suggesting it is a basic protein. The hydrophilicity of LACV-mVax01 is demonstrated by its predicted GRAVY score of −0.147, reflecting its solubility in water. Despite this hydrophilic nature, LACV-mVax01 is stable, with an instability index of 17.12 (Table 1).

**Table 1:** The physicochemical and immunological features of the LACV-mVax01.

Physicochemical features	Value
Molecular Weight (Da)	47189.42
Number of amino acids	425
Theoretical pI	8.99
GRAVY	−0.147
Instability index	17.12
Aliphatic index	81.88
Total number of negatively charged residues (Asp + Glu)	50
Total number of positively charged residues (Arg + Lys)	59
Number of atoms	6697
Solubility (SOSUI/ SOLpro)	Soluble protein
**Immunological features**	
Allergenicity (AllerTOP v. 2.0/ AllergenFP v.1.0/ AlgPred)	Non-allergen
Antigenicity (VaxiJen 2.0/ ANTIGENpro)	Antigen
Toxicity (ToxinPred)	Non-toxin

Additionally, distinct characteristics of LACV-mVax01 include its hydrophobic nature and aliphatic side chains, supported by an aliphatic index of 81.88. The protein is also characterized by more positively charged residues (59) than negatively charged residues (50). The SOSUI server suggested its solubility, assigning a hydrophobicity score of −0.147. Additionally, the SOLpro server yielded a high solubility score of 0.879293, indicating that LACV-mVax01 can be successfully expressed in *E. coli*. Immunological assessment of LACV-mVax01 further validated its safety and efficacy. Tools such as AllerTOP version 2.0, AllergenFP version 1.0, and AlgPred suggested the absence of allergic reactions in recipients. Moreover, the ANTIGENpro, VaxiJen 2.0, and ToxinPred servers identified LACV-mVax01 as a potential antigen and non-toxic protein, underscoring its promise as a vaccine candidate (Table 1).

### 3.6. Structural prediction and validation

The GOR4, SOPMA, and PSIPRED servers were used to estimate the two-dimensional structure of the LACV-mVax01. The PSIPRED server used a three-state method to predict the two-dimensional structure of LACV-mVax01 and also provided spatial and polar attributes for individual amino acids. Although the SOPMA server suggested that the two-dimensional structure of LACV-mVax01 would consist of an alpha helix, random coils, and extended strands, the actual proportions of these components were 49.41%, 11.06%, and 32.47%, respectively. In the GOR4 server, the proportions of alpha helices, random coils, and extended strands (beta sheets) were 48.94%, 33.41%, and 17.65%, respectively (S4 Table and S4 Fig).

**Fig 3:**
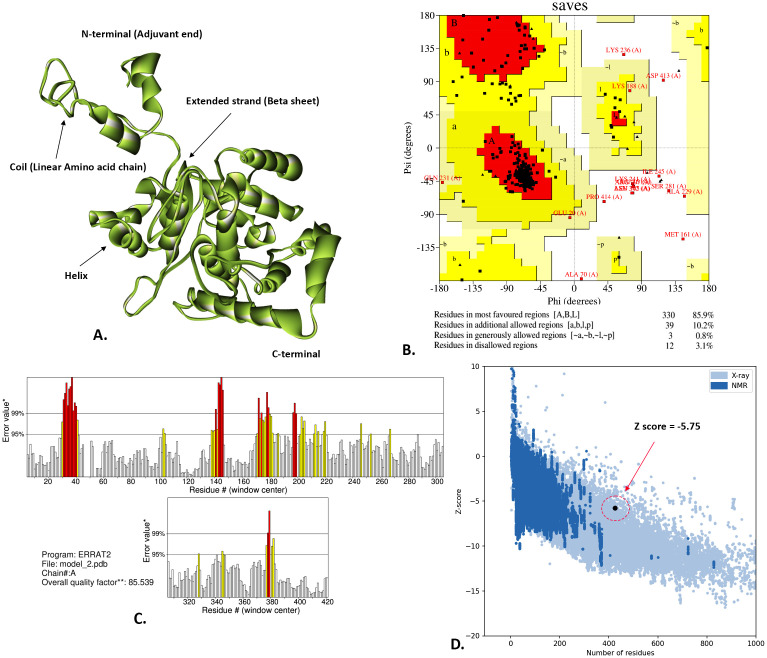
Validation of the I-TASSER predicted model of the LACV-mVax01 after Ramachandran plot (A), Z-score (B), and ERRAT (C), and Ribbon 3D model (D).

The I-TASSER server provided five unique models, and the first was selected for its desirable TM score of 0.49 ± 0.15, C-score of −1.86, and RMSD of 11.3 ± 4.5 Å. Following that, the refined model of LACV-mVax01 was utilized for the subsequent study. This model had an RMSD value of 0.489, a MolProbity of 2.230, a clash score of 12.5, a bad rotamer score of 0.9, and a rama-preferred score of 87.0. In light of these findings, LACV-mVax01 can be considered a better model. According to the Ramachandran plot of the SAVES server, the energy-minimized model of LACV-mVax01 contained 85.9% of its amino acid residues in the most preferred areas, 10.2% to the allowed regions, and 0.8% to the generously allowed regions. The refined LACV-mVax01 model had an ERRAT score of 85.539. Additionally, the refined model of LACV-mVax01 was validated by the ProSA server, which achieved a Z-score of −5.75 KJ/mol (Fig 3).

### 3.7. Prediction of CBL epitopes

A total of 232 CBL epitope residues were identified using the Discotope 2.0 server. The LACV-mVax01 contains 425 amino acids, and the epitope scores vary from 0.508 to 0.843, subject to the number of residues involved (S5 Table).

### 3.8. Molecular docking analysis of the LACV-mVax01 with the TLRs

**Fig 4:**
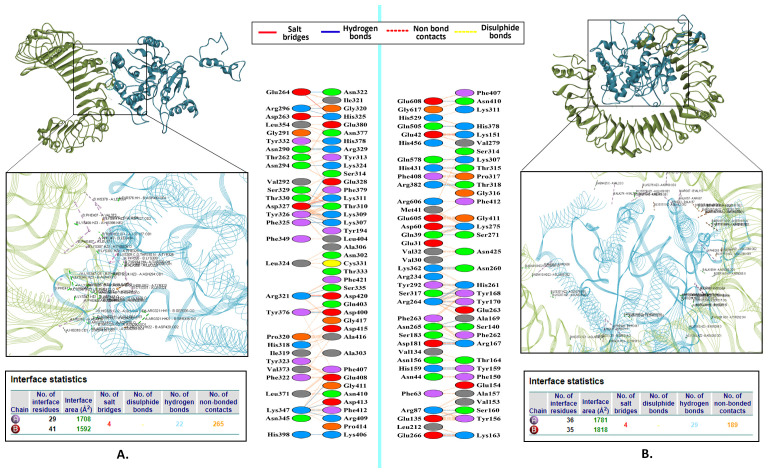
The interaction between LACV-mVax01-TLR2 (A) and LACV-mVax01-TLR4 (B) complexes. The vaccine and receptors are shown in blue and olive colors, respectively, while their interactions are illustrated with varied hues that reflect the characteristics of the bonds.

The docking analysis of human TLR2 and TLR4 receptors with LACV-mVax01 was performed using the CLUSPRO 2.0 server. For the LACV-mVax01-TLR2 complex, the selected docked model had the lowest energy score of −939.2 and a center energy score of −902.7. Where the chosen model for the LACV-mVax01-TLR4 complex exhibited the lowest energy score of −1000.0 and a center energy score of −764.8. The LACV-mVax01-TLR2 complex demonstrated a total of 265 non-bond interactions, including 22 hydrogen bonds and 4 salt bridges. In comparison, the LACV-mVax01-TLR4 complex showed 189 non-bond interactions, 29 hydrogen bonds, and 4 salt bridges (Table 2 and Fig 4).

**Table 2:** The docking scores and interactions between the LACV-mVax01-TLR complexes.

Complex	Weighted score		Molecular Interactions			
	Lowest energy	Center	Salt bridges	Disulphide bonds	Hydrogen bonds	Non-bonded contacts
LACV-mVax01-TLR2	−939.2	−902.7	4	–	22	265
LACV-mVax01-TLR4	−1000.0	−764.8	4	–	29	189

### 3.9. Molecular dynamics simulation and post-simulation analysis

#### 3.9.1. RMSD analysis.

**Fig 5:**
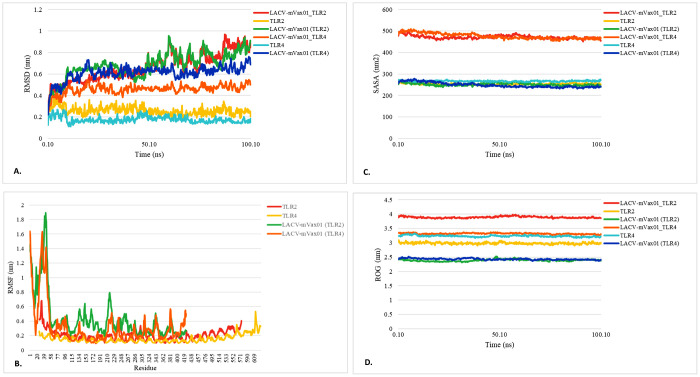
The post-simulation analyses including RMSD (A), RMSF (B), SASA (C) and ROG (D) of the LACV-mVax01 and its TLR complexes.

The RMSD trajectories of the LACV-mVax01_apo, LACV-mVax01_TLR2, and LACV-mVax01_TLR4 complexes were evaluated to validate the stability of the docked complexes (Fig 5A). The RMSD values for the LACV-mVax01_apo complex showed minimal fluctuation during the simulation. At the 100 ns trajectory, the average RMSD was 0.71 nm, suggesting relatively stable structural behavior throughout the simulation. The LACV-mVax01_TLR2 complex, on the other hand, showed slightly distinct RMSD dynamics. While the individual components, TLR2 and LACV-mVax01, exhibited average RMSD values of 0.26 nm and 0.68 nm, respectively, the overall complex had an average RMSD of 0.66 nm (Fig 5A). In the comparison of LACV-mVax01_apo and LACV-mVax01_TLR2, the RMSD of LACV-mVax01 exhibited a more variable pattern in the complex state than in its apo form (Figs 5A and S5A). After the 50 ns simulation, the RMSD values of LACV-mVax01_apo decreased, whereas those of LACV-mVax01 in the TLR2 complex increased. In the LACV-mVax01_TLR4 simulation, the average RMSD at 100 ns was determined to be 0.56 nm, with the average RMSD values for TLR4 and LACV-mVax01 being 0.19 nm and 0.75 nm, respectively. In the comparison of LACV-mVax01_apo and LACV-mVax01_TLR4, the RMSD of LACV-mVax01 exhibited a more variable pattern in the complex form than in its apo state. After the 50 ns simulation, the RMSD values for LACV-mVax01_apo showed a declining trend. In comparison, LACV-mVax01 docked with the TLR2 exhibited an abrupt peak of about 24 ns and continued to vary until the end of the simulation (Figs 5A and S5A). The LACV-mVax01 within the LACV-mVax01–TLR2 complex demonstrated comparatively improved binding stability relative to the vaccine apo structure, as indicated by lower average RMSD values. This suggests that the interaction between LACV-mVax01 and TLR2 may contribute to the stabilization of the complex. The LACV-mVax01-TLR4 exhibited a more stable structure, with a lower average RMSD than LACV-mVax01_apo. Furthermore, during the simulation, the RMSD values for each compound exhibited less fluctuation in the LACV-mVax01_TLR4 compared to those in the LACV-mVax01_TLR2.

#### 3.9.2. RMSF analysis.

The RMSF values of each amino acid residue in the LACV-mVax01_apo, LACV-mVax01_TLR2, and LACV-mVax01-TLR4 complexes were calculated to evaluate residue-level stability and interaction dynamics. Upon completion of the simulation (100 ns), the average residual RMSF values for LACV-mVax01_apo were estimated to be 0.28 nm. In the LACV-mVax01_TLR2, the average RMSF values for each residue were 1.36 nm, but the RMSF for the equivalent residues of TLR2 and LACV-mVax01 were 1.33 nm and 1.40 nm, respectively (Figs 5B and S5B). During the simulation, the residues of LACV-mVax01 within LACV-mVax01_TLR2 showed much more significant variations than those in other states. While comparing the RMSF values with LACV-mVax01_apo, it was observed that the residues in LACV-mVax01 exhibited more fluctuation and instability when bound to TLR2. The average RMSF values for TLR4 and LACV-mVax01 in the LACV-mVax01_TLR4 complex were 0.12 nm and 0.24 nm, respectively, leading to a total complex RMSF of 0.16 nm (Figs 5B and S5B). The low RMSF values reflected minimal residue fluctuations, which may indicate overall structural stability throughout the simulation period. When comparing the RMSF profiles of LACV-mVax01 across different states, the residues of LACV-mVax01 showed relatively lower fluctuations in the TLR2-bound form than in the unbound (LACV-mVax01_apo) state. A similar trend was observed in the TLR4-associated complex, where fluctuations appeared further reduced. The comparatively lower RMSF values in the LACV-mVax01–TLR4 complex, relative to the LACV-mVax01–TLR2 complex, may indicate a tendency toward reduced structural fluctuations during interaction with TLR4.

#### 3.9.3. SASA analysis.

The SASA values of each complex were assessed to elucidate protein folding, stability, and interaction dynamics, since solvent infiltration into the hydrophobic core may affect structural integrity and functionality. A low SASA value indicates that the hydrophobic core primarily prevents water from contacting the protein, whereas a high value indicates that the protein is significantly exposed to water. The current study also assessed the SASA values of several LACV-mVax01 complexes, including LACV-mVax01_apo, LACV-mVax01_TLR2, and LACV-mVax01-TLR4, to analyze their structural stability and interactions. Following the 100 ns simulation, the average SASA value for the LACV-mVax01_apo complex was determined to be 243.30 nm². For the LACV-mVax01_TLR2 complex, the average SASA values were 253.46 nm² for TLR2 and 244.52 nm² for LACV-mVax01. The total SASA value for the LACV-mVax01_TLR2 complex was notably elevated, averaging 471.81 nm². Therefore, the LACV-mVax01 in the LACV-mVax01_TLR2 complex exhibited a higher SASA value compared to the LACV-mVax01_apo structure, suggesting a potential structural expansion or increased solvent exposure when bound to TLR2. In the LACV-mVax01_TLR4 complex, the average SASA values for TLR4 and LACV-mVax01 were determined to be 267.89 nm^2^ and 247.21 nm^2^, respectively. Nonetheless, the LACV-mVax01_TLR4 complex exhibited an average SASA value of 472.22 nm^2^ (Figs 5C and S5C). The analysis revealed that the LACV-mVax01 in the LACV-mVax01_TLR4 complex exhibited a higher SASA than the LACV-mVax01_apo structure, indicating increased solvent exposure upon binding to TLR4. Nevertheless, a significant difference was observed in the SASA values of LACV-mVax01 in complexes with TLR2 and TLR4. The SASA value of LACV-mVax01 was significantly higher when bound to TLR2 than to TLR4.

#### 3.9.4. ROG analysis.

The ROG is an approach used to assess a protein’s compactness. For a protein’s folding to be deemed stable, the ROG value must be comparatively constant. Biological systems with reduced ROG profiles have more flexibility, contrary to the characteristics of other biological systems. This post-simulation study assessed the ROG values for several structures, including LACV-mVax01_apo, LACV-mVax01_TLR2, and LACV-mVax01_TLR4. At the end of the simulation (100 ns), the average ROG value of the LACV-mVax01_apo structure was found to be 2.34 nm. In the LACV-mVax01_TLR2 complex, the average ROG values of TLR2 and LACV-mVax01 were 2.98 nm and 2.39 nm, respectively. The complex itself exhibited a slightly higher ROG value of 3.89 nm, indicating that LACV-mVax01 was more flexible in the bound state with TLR2 compared to its apo structure. In contrast, the analysis of the LACV-mVax01_TLR4 complex revealed different ROG dynamics. The average ROG values of TLR4 and LACV-mVax01 were 3.21 nm and 2.39 nm, respectively (Figs 5D and S5D). The complex had a ROG value of 3.28 nm, indicating that LACV-mVax01 was less flexible when bound to TLR4 than in its unbound apo form. When comparing the two vaccine-receptor complexes, the LACV-mVax01_TLR2 complex displayed greater flexibility than the LACV-mVax01_TLR4 complex. This was reinforced by changes in ROG values, which indicated distinct structural features depending on the receptor they bind to.

#### 3.9.5. Hydrogen bonds analysis.

**Fig 6:**
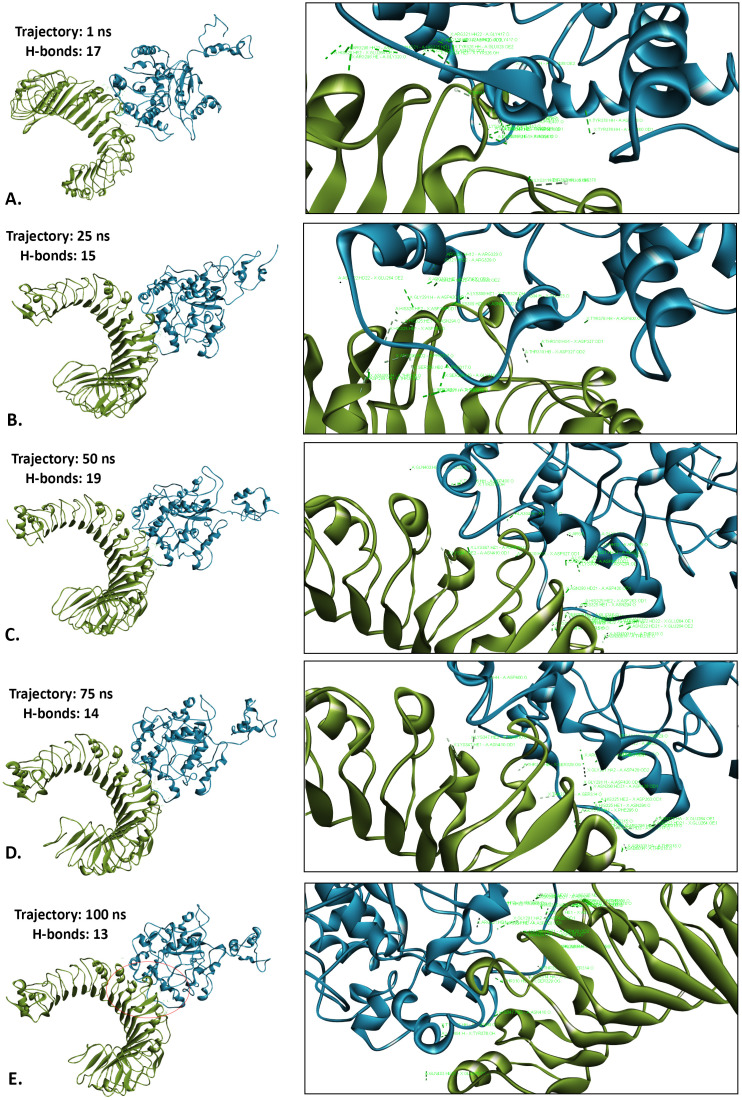
The H-bond analysis of the LACV-mVax01_TLR2 complex at different simulation trajectories including 1 ns (A), 25 ns (B), 50 ns (C), 75 ns (D) and 100 ns (E).

**Fig 7:**
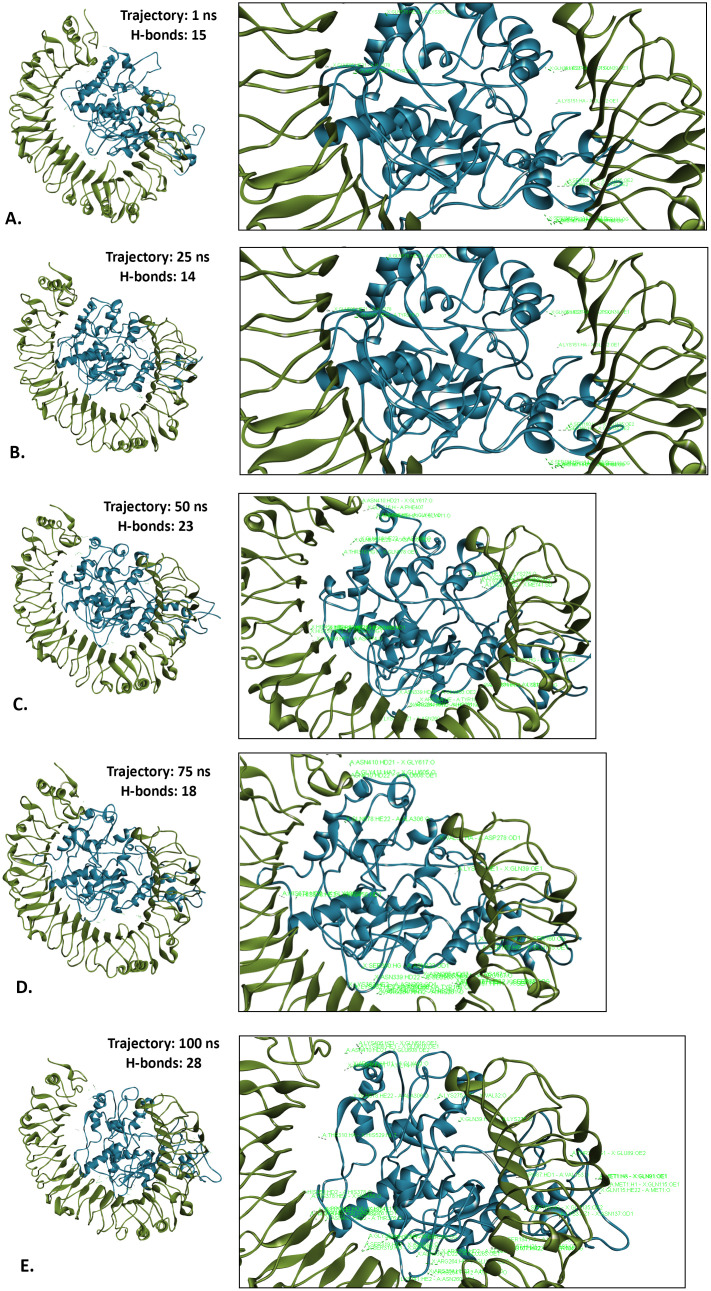
The H-bond analysis of the LACV-mVax01_TLR4 complex at different simulation trajectories including 1 ns (A), 25 ns (B), 50 ns (C), 75 ns (D) and 100 ns (E).

Hydrogen bonds are crucial for molecular recognition, providing precise, directed interactions between proteins and their ligands. As a post-simulation analysis, the number of hydrogen bonds was evaluated for the LACV-mVax01-apo, LACV-mVax01_TLR2 and LACV-mVax01_TLR4 at different simulation trajectories. At the beginning of the simulation run (1 ns trajectory), the number of hydrogen bonds was 17 and 15 for LACV-mVax01_TLR2 and LACV-mVax01_TLR4, respectively (S5 Fig). Within the LACV-mVax01_TLR2 10 (GLU-264, ASN-294, ARG-296, ARG-321, TYR-323, PHE-325, TYR-326, ASP-327, TYR-332, LYS-347) and 13 (LYS-307, THR-310, LYS-311, GLY-320, LYS-325, HIS-325, ASP-400, PHE-407, GLU-408, ASN-410, PHE-412, GLY-417, ASP-420) amino acid residues were found to be form hydrogen bonds from the TLR2 and LACV-mVax01 respectively (Figs 6A and S6A). While the LACV-mVax01_TLR4 had 11 (LYS-151, TYR-156, LYS-163, ARG-167, ASN-260, HIS-261, LYS-275, SER-314, THR-318, ASN-377, HIS-378) and 13 (GLU-42, ASP-60, GLU-135, ASP-181, ARG-264, GLU-266, ASP-294, LYS-362, ARG-382, HIS-431, GLY-480, GLN-505, GLN-507) amino acid residues to be form hydrogen bonds from the LACV-mVax01 and TLR4 respectively (Figs 7A and S6B).

At the 25 ns simulation trajectory, the number of hydrogen bonds was found to be 15 for the LACV-mVax01_TLR2 complex and 14 for the LACV-mVax01_TLR4 complex (S6 Fig). In the LACV-mVax01_TLR2 interaction, 11 residues from the TLR2 (ASP-263, GLU-264, ASN-294, ALA-297, SER-298, ASN-300, ARG-321, TYR-323, TYR-326, ASP-327, TYR-376) and 12 residues from the LACV-mVax01 (ALA-304, LYS-309, THR-310, LYS-311, GLY-316, THR-318, ASN-322, HIS-325, GLU-328, ARG-329, ASP-400, and ASP-420) were involved in hydrogen bond formation (Figs 6B and S6A). For the LACV-mVax01_TLR4 complex, 11 residues from the TLR4 (GLN-39, ARG-87, GLU-135, SER-183, SER-184, ARG-264, GLU-266, LYS-362, GLN-505, GLN-578, GLU-608) and 9 residues from the LACV-mVax01 (SER-160, LYS-163, ARG-167, TYR-168, GLU-263, LYS-275, LYS-307, TYR-313, HIS-378) were identified in hydrogen bond formation (Figs 7B and S6B).

At the 50 ns simulation trajectory, the number of hydrogen bonds observed was 19 for LACV-mVax01_TLR2 and 23 for LACV-mVax01_TLR4 (S6 Fig). In the LACV-mVax01_TLR2 complex, 12 amino acid residues from the TLR2 (ASP-263, GLU-264, ASN-290, ALA-297, SER-298, ASN-300, ARG-321, TYR-323, ASP-327, LYS-347, GLU-375, TYR-376) and 14 from the LACV-mVax01 (ALA-304, THR-310, LYS-311, THR-315, GLY-316, THR-318, ASN-322, HIS-325, ARG-329, ASP-400, GLN-403, LEU-404, ASN-410, ASP-420) formed hydrogen bonds (Figs 6C and S6A). In the LACV-mVax01_TLR4 complex, 17 residues from the TLR4 (GLN-39, ASP-60, GLU-135, SER-183, SER-184, ARG-264, GLU-266, ASN-339, LYS-341, LYS-362, HIS-431, HIS-458, GLN-578, ARG-606, GLU-608, GLN-616, GLY-617) and 15 from the LACV-mVax01 (SER-160, LYS-163, ARG-167, TYR-168, ASN-260, HIS-261, GLU-263, LYS-275, LYS-307, THR-310, THR-375, THR-376, ASP-400, ASN-410, GLY-411) formed hydrogen bonds (Figs 7C and S6B).

At the 75 ns simulation trajectory, the LACV-mVax01_TLR2 complex revealed 14 hydrogen bonds, whereas the LACV-mVax01_TLR4 complex had 18 hydrogen bonds (S6 Fig). In the LACV-mVax01_TLR2 complex, 10 amino acid residues from the TLR2 (ASP-235, ASP-263, GLU-264, ASN-290, GLY-291, ASN-294, ALA-297, ASN-300, ARG-321, TYR-376) and 8 residues from the LACV-mVax01 (THR-315, THR-318, ASN-322, HIS-325, GLU-328, ARG-329, ASP-400, ASP-420) were involved with hydrogen bond formation (Figs 6D and S6A). In the LACV-mVax01_TLR4 complex, 13 residues from the TLR4 (GLU-42, ARG-87, GLU-135, SER-183, SER-184, ARG-264, GLU-266, ASN-339, SER-360, HIS-456, GLN-578, GLU-608, GLY-617) and 12 residues from the LACV-mVax01 (LYS-151, SER-160, LYS-163, ARG-167, TYR-168, HIS-261, GLU-263, ALA-306, LYS-307, ASN-322, HIS-378, ASN-410) were recognized as hydrogen bonds forming amino acids (Figs 7D and S6B).

At the 100 ns trajectory, the number of hydrogen bonds was determined to be 13 for LACV-mVax01_TLR2 and 28 for LACV-mVax01_TLR4 (S6 Fig). In the LACV-mVax01_TLR2, a total of 10 amino acid residues (ASP-235, ASP-263, ASN-290, GLY-291, ASN-294, ALA-297, ASN-300, ARG-321, LYS-347, TYR-376) were identified as forming hydrogen bonds with the TLR2, while 8 residues (THR-315, THR-318, HIS-325, ARG-329, ASP-400, THR-410, ASP-420, LYS-424) were noted for the LACV-mVax01 (Figs 6E and S6A). The LACV-mVax01-TLR4 exhibited 11 amino acid residues (VAL-32, GLN-39, GLU-42, GLU-89, GLN-91, GLN-115, GLU-135, ASN-137, SER-184, ARG-264, GLU-266) capable of forming hydrogen bonds with the TLR4, while the LACV-mVax01 demonstrated 12 residues (MET-1, ALA-2, LYS-3, THR-35, LYS-151, SER-160, LYS-163, ARG-167, TYR-168, HIS-261, GLU-263, LYS-275) were involved in this bond formation (Figs 7E and S6B).

#### 3.9.6. MMPBSA.

The LACV-mVax01_TLR2 complex had scores of −7520.53 kcal/mol for ΔVDWAALS, −70997.86 kcal/mol for ΔEEL, −11308.62 kcal/mol for ΔEGB, and 339.43 kcal/mol for ΔESURF. These interactions resulted in a total binding (ΔTOTAL) free energy of −20524.57 kcal/mol (Table 3). Within the LACV-mVax01_TLR4 complex, the anticipated MMPBSA values for ΔVDWAALS, ΔEEL, ΔEGB, and ΔESURF were – 8143.47 kcal/mol, −75104.82 kcal/mol, −11389.22 kcal/mol, and 340.65 kcal/mol, respectively, with a total binding free energy of −20868.10 kcal/mol (Table 3).

**Table 3:** The MMPBSA analysis of the LACV-mVax01_TLR2 and LACV-mVax01_TLR4.

Complex	ΔVDWAALS (kcal/mol)	ΔEEL (kcal/mol)	ΔEGB (kcal/mol)	ΔESURF (kcal/mol)	ΔTOTAL (kcal/mol)
LACV-mVax01_TLR2	−7520.53	−70997.86	−11308.62	339.43	−20524.57
LACV-mVax01_TLR4	− 8143.47	−75104.82	−11389.22	340.65	−20868.10

### 3.10. Optimization of codon and virtual molecular cloning

**Fig 8:**
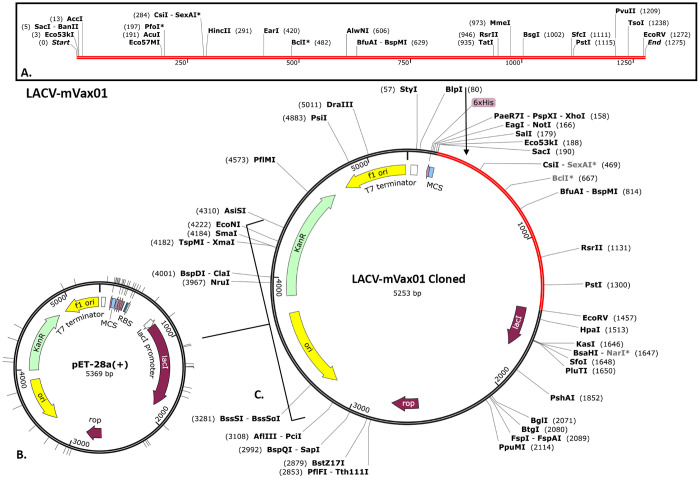
Cloning of the LACV-mVax01 (A) using the pET-28a(+) plasmid vector (B). The expression of pET-28a(+), a plasmid vector linked to the LACV-mVax01 sequence, was enabled via the SnapGene software, with the red section indicating the gene coding for the vaccine and the black circle denoting the vector backbone (C).

The 1250-nucleotide coding sequence of LACV-mVax01 was previously available on the JCat website prior to the cloning efforts. With an average GC content of 46.90% and a CAI of 0.9848, the modified coding sequence exhibited high-level expression in the *E. coli* vector. The coding sequences were inserted into the pET-28a(+) plasmid vector using the SnapGene software and the recombinant plasmid sequence (Fig 8).

### 3.11. Immune simulation and in silico cloning

**Fig 9:**
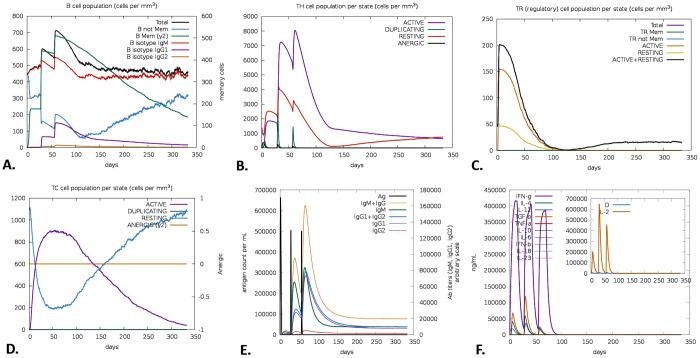
C-ImmSim predicted an immune simulation of the LACV-mVax01, including the evolution of B-cell populations (A), TH cells (B), TR cells (C), TC cells (D), as well as immunoglobulins (E) and cytokines (F) responses.

Following three vaccine doses, an increase in the proportion of memory B-cells within the total B-cell population was observed. The B-cell isotype IgM was observed at higher levels compared to IgG1 and IgG2. Continuous monitoring of total B-cell counts suggested a sustained immune response over the one-year observation period (Fig 9A). Following the immunization schedule, increases in T-cell responses were observed, including T helper (TH), T regulatory (TR), and cytotoxic T cells (TC) (Fig 9B–D). On day 60, relatively higher levels of active and memory TH cells were detected, which were maintained for approximately one month before gradually declining. Active Treg and TC populations were also observed during the post-vaccination period and remained detectable throughout the one-year follow-up. On day 60, elevated levels of IgM + IgG antibodies were recorded; these levels subsequently declined but remained measurable for up to one year (Fig 9E). Additionally, increased production of interferon-gamma (IFN-γ), along with moderate levels of transforming growth factor beta (TGF-β), interleukin-2 (IL-2), and interleukin-12 (IL-12), was observed during the study period (Fig 9F), consistent with ongoing immune activation.

### 3.12. mRNA structure prediction

**Fig 10:**
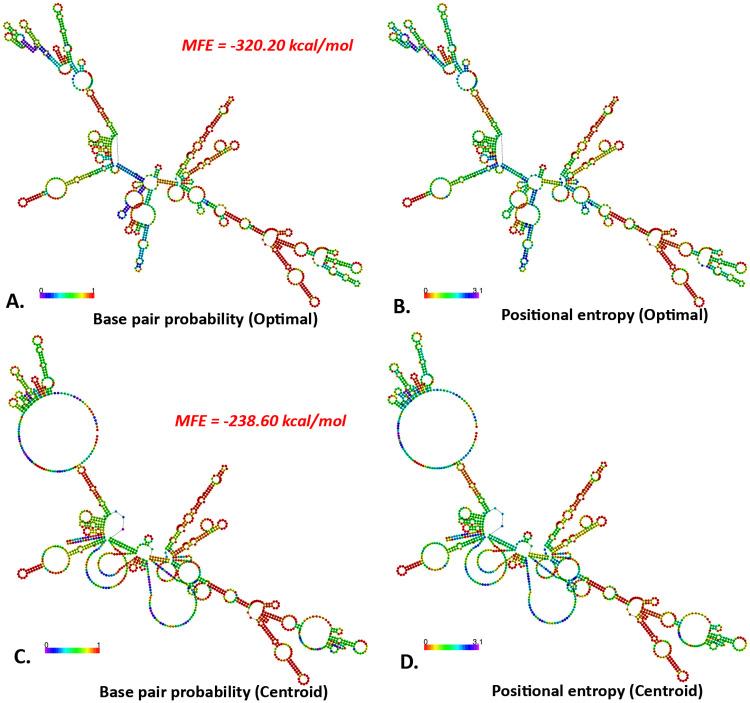
Predicted mRNA structure of the LACV-mVax01 by RNAfold web server. The centroid structures of the LACV-mVax01 with the base pair probabilities (A and C) and the positional entropy (B and D).

The two-dimensional structure of LACV-mVax01 has been identified with an MFE score of −320.20 kcal/mol for the optimal structure and −238.60 kcal/mol for the centroid structure. The thermodynamic ensemble was expected to have a value of −342.16 kcal/mol. Furthermore, the ensemble for the MFE structure of LACV-mVax01 exhibited a correlation of 0.00%. Thus, the proposed vaccine’s mRNA structure will be robust and effective during its entry, transcription, and expression in the host (Fig 10).

## 4. Discussion

The LACV has become the predominant cause of arboviral disease globally, especially in the US. Although the majority of LACV infections are asymptomatic or lead to mild febrile illness, neuroinvasive cases were seen in children under the age of 16 [80]. In critical instances, intravenous fluids are provided, but in reduced volumes, to mitigate the risk of symptomatic cerebral edema. Developing a vaccine for LACV would signify a remarkable advancement in combating the disease. The advent of recombinant DNA and whole-genome sequencing technologies revolutionized vaccine research, enabling the development of novel pathogen-specific vaccines [81]. In recent decades, vaccination has become a significant public health concern due to outbreaks of infectious diseases such as measles and coronavirus disease-19 (COVID-19) [82]. Animal experiments have been performed to explore immunization against LACV; however, no human clinical trials have been completed yet [18]. Nonetheless, immunizing newborn BALB/c mice with recombinant G1 protein induces efficient immunity, whereas mice with a targeted disruption in the ß-chain of the IFNAR-1 receptor demonstrate heightened susceptibility to LACV [83,84]. Meanwhile, reverse vaccinology (RV) is a technique that employs immunoinformatics and *in silico* assessment of pathogen genomes to swiftly and precisely identify potential vaccine targets [85–87]. Following the advent of RV, the first two COVID-19 vaccines (mRNA-1273 and BNT162b2) were developed in less than a year [88–91].

In this study, a multiepitope vaccine was designed to combat LACV infection in humans. A crucial aspect is that the vaccine can prevent infections associated with multiple strains of LACV. The epitopes chosen for the vaccine are selected based on their ability to stimulate cytokine production while being non-allergenic, non-toxic, and antigenic. The addition of the bL12 adjuvant appeared to contribute to enhanced vaccine responses [92]. Combining MHC-I (98.55%) and MHC-II (99.99%) epitopes, the IEDB server showed broad worldwide population coverage reaching 100%. Except for South Africa (95.27%), the regional study revealed almost uniform coverage throughout the continents. The LACV-mVax01 candidate was deemed suitable for vaccination owing to its molecular weight of 47189.42 Da and its stability in biological contexts, as evidenced by an instability score of 17.12; molecules with an instability index below 40 are considered stable [93,94]. The vaccine also exhibited hydrophobic properties, with an aliphatic index of 81.88. Understanding how proteins fold into their two and three-dimensional shapes is crucial for developing effective vaccines. Unfolded protein regions and α-helical coils have substantial impacts on protein-specific immune responses. The structural proteins can be readily refolded into their original conformation, making them highly susceptible to antibody responses during infections. Both the GOR4 and SOPMA servers projected the two-dimensional structure of the LACV-mVax01 to consist of an alpha helix at 49.65% and 40.00%, respectively [74,95]. The substantial amount of beta sheets, coils, and alpha helices in the secondary structure may contribute to its overall stability.

Following the tertiary structure prediction, model 1 was chosen with a TM-score of 0.49 ± 0.15 and a C score of −1.86. A higher TM score implies a greater analogy, and the score falls within the range of (0, 1]. The C-score, ranging from −5–2, signifies a highly assured model, whereas a lower value suggests a less compact structure [96]. The LACV-mVax01 model was then optimized using GalaxyRefine, and the second model was selected based on its RMSD value of 0.489, MolProbity score of 2.230, and Ramachandran score of 87.0%. A high MolProbity score indicates that the vaccine model is more reliable due to fewer steric conflicts and better overall geometry [97]; the low RMSD aids in determining the extent to which the projected structure aligns with a reference structure [98]. Regarding the Ramachandran score, structures with a high number of residues in favorable regions are generally more stable and have functional relevance [99,100]. The Ramachandran plot analysis of SAVES revealed that a significant proportion (85.9%) of the residues were in the most favorable positions. The ERRAT score reflects the quality of non-bonded atomic interactions, with higher values suggesting a tendency toward better structural reliability. Typically, a model is deemed superior if it falls within the acceptable threshold of 50 or higher [101]. The Z-score of a predicted model structure indicates the model’s correctness and potential flaws, with higher negative values indicating higher quality [102,103]. Hence, the LACV-mVax01 was validated with a Z-score of −5.75 kJ/mol and an ERRAT score of 85.539, indicating its quality. A docking analysis utilizing TLR2 and TLR4 was conducted to determine the potential for binding interactions between the LACV-mVax01 and immune cells. The docking analysis showed a lower energy score for the LACV-mVax01–TLR4 complex (−1000.0) compared to the LACV-mVax01–TLR2 complex (−939.2), which may suggest relatively stronger binding with TLR4. The interaction analysis showed that the TLR2 complex had 265 non-bond interactions, 22 hydrogen bonds, and 4 salt bridges. Compared with the TLR4 complex, the TLR4 complex had 189 contacts, 29 hydrogen bonds, and 4 salt bridges.

Further, MMPBSA analysis indicated that the LACV-mVax01–TLR4 complex had a slightly lower total binding free energy (−20868.10 kcal/mol) compared to the LACV-mVax01–TLR2 complex (−20524.57 kcal/mol), which may reflect a modest increase in stability. The primary contributions came from ΔVDWAALS and ΔEEL energies, with TLR4 values (−8143.47 and −75104.82 kcal/mol) marginally higher than those of TLR2 (−7520.53 and −70997.86 kcal/mol) [104]. In molecular dynamics simulation, the RMSD analysis monitors the average distance between the backbone atoms of a protein or nucleic acid and a reference structure over time. The low RMSD values suggest that the structure of LACV-mVax01 remained relatively stable with minimal conformational changes. Over time, RMSF calculates the average deviation of atoms from their original positions. The presence of low RMSF values indicates that atoms remain in their usual places, which indicates that there is little flexibility [105]. The LACV-mVax01–TLR2 complex showed relatively stable binding, with an average RMSD of 0.66 nm, while the LACV-mVax01–TLR4 complex exhibited slightly lower RMSD values (0.56 nm). RMSD fluctuations were generally smaller in the TLR4 complex compared to TLR2, suggesting a possible trend toward more stable interactions with TLR4. The LACV-mVax01–TLR2 complex showed larger fluctuations (1.36 nm) than the TLR4 complex (0.16 nm), indicating comparatively lower structural variability in the latter. Compared with the apo form (0.28 nm), residues of LACV-mVax01 displayed reduced fluctuations in both complexes, with the TLR4 complex showing the lowest overall RMSD during the simulation.

The SASA values were evaluated to make a more accurate prediction regarding the amount of solvent that would reach the hydrophobic center of a protein. A higher SASA value indicates that a significant portion of the protein’s surface area is exposed to the solvent, which might increase its solubility. The ability of proteins to dissolve in water is an important feature that dictates how efficiently they function in biological systems [106]. The SASA study indicated increased solvent exposure of LACV-mVax01 within both the TLR2 and TLR4 complexes relative to its apo form (243.30 nm²). The LACV-mVax01_TLR2 complex exhibited a larger solvent-accessible surface area (SASA) of 244.52 nm² compared to TLR4’s 247.21 nm², indicating greater structural expansion with TLR2 and underscoring differences in interaction kinetics and receptor binding stability. By analyzing the ROG, one can infer a molecule’s overall structural quality and compactness level. A lower ROG points to a more compact structure, whereas a higher ROG indicates an expanded form. The LACV-mVax01–TLR2 exhibited a somewhat higher ROG (3.89 nm) compared to LACV-mVax01–TLR4 (3.28 nm), consistent with slightly increased conformational variability. Compared with the apo state (2.34 nm), LACV-mVax01 showed relatively higher flexibility when bound to TLR2 and lower flexibility with TLR4, reflecting differences in structural dynamics between the two receptor complexes. The structural integrity of macromolecules such as complex polysaccharides, nucleic acids, and proteins is maintained by hydrogen bonding. For molecular buildings, they aid in stabilization and correct folding [107]. Following simulation of LACV-mVax01 complexes with TLR2 and TLR4, the analysis revealed dynamic hydrogen-bond interactions along multiple pathways. At 1 ns, the LACV-mVax01_TLR2 formed 17 hydrogen bonds, whereas the LACV-mVax01_TLR4 formed 15. At 50 ns, the number of hydrogen bonds increased to 19 in the LACV-mVax01_TLR2 and 23 in the LACV-mVax01_TLR4. At 100 ns, the LACV-mVax01_TLR4 exhibited the highest hydrogen bond count, reaching 28, which surpassed the 13 observed for the LACV-mVax01_TLR2. Residue-level analysis identified the amino acids involved in hydrogen bond formation in each complex, with the LACV-mVax01–TLR4 complex showing a tendency toward more stable interactions compared to LACV-mVax01–TLR2. The expression profile of recombinant LACV-mVax01 in the *E. coli* K12 strain was assessed through virtual codon optimization. The vector showed a relatively high potential for expression, with a GC content of 46.90% and a CAI score of 0.9848. Following the vaccination regimen, immune responses were observed over the study period. Memory B cells showed an upward trend, with IgM responses being more pronounced than IgG isotypes. Memory TH cells were relatively elevated for approximately one month, and T-cell populations, including TH, TR, and TC cells, peaked around day 60 post-vaccination. IgM + IgG antibody levels increased after vaccination but gradually declined over the course of the year, remaining detectable throughout the follow-up period. Cytokine profiling indicated moderate levels of TGF-β, IL-2, and IL-12, alongside elevated IFN-γ, consistent with ongoing immune activity and suggesting a sustained, though gradually decreasing, immune response following vaccination. Regarding mRNA structure, the structural integrity of the LACV-mVax01 was validated by MFE scores of −238.60 kcal/mol and −320.20 kcal/mol for centroid and optimal structures, respectively. This phenomenon was further validated by the thermodynamic free energy of −342.16 kcal/mol. Notably, the vaccines’ MFE structures show a 0.00% frequency in the ensemble, highlighting their steady stability, as a negative MFE score indicates the stability of an mRNA molecule [108]. Consequently, LACV-mVax01’s mRNA is optimal for host entrance, transcription, and expression, ensuring its effectiveness and usefulness. This research indicates that the LACV-mVax01 is effective and may elicit substantial humoral and cellular immune responses by targeting critical epitopes of the G1, G2, and N proteins. However, this work requires validation of its results through *in vitro*, clinical, and *in vivo* evaluations.

## 5. Conclusion

This study employed immune informatics and reverse vaccinology to design a novel multiepitope vaccine, LACV-mVax01, targeting LACV infection. The vaccine incorporates consensus sequences from essential proteins, which may help provide protection against multiple LACV strains. The construct was predicted through computational analyses to have antigenic potential while showing no apparent toxicity or allergenic properties. Immunological simulations indicated likely activation of T-cells and B-cells, as well as IFN-γ production. Structural analysis supported the vaccine’s stability, and molecular docking demonstrated effective binding to TLR-2 and TLR-4 receptors. Molecular dynamics simulations further supported the binding affinity, stability, and solvent accessibility of the vaccine-receptor complexes. Additionally, the mRNA structure exhibited high energy scores, indicating probable stability during cellular entry, transcription, and expression. Overall, LACV-mVax01 demonstrated predicted antigenicity and the capacity to elicit immune responses in silico. These findings may inform future vaccine development and antiviral strategies against LACV, pending experimental validation.

### 5.1. Limitation of the study

The findings of this study are based solely on *in silico* analyses and therefore constitute preliminary predictions rather than validated biological outcomes. The lack of *in vitro* binding assays, experimental expression and stability testing of the vaccine construct, and *in vivo* validation in animal models limits the reliability of the biological interpretation. Therefore, the proposed vaccine candidate remains a theoretical model that necessitates comprehensive laboratory experimentation. Subsequent research should involve experimental synthesis, structural and stability assessments, receptor-binding validation, and immunogenicity testing in appropriate biological systems to determine the actual protective efficacy of the construct.

## Supporting information

S1 Fig Multiple sequence alignment of the G1 proteins sequences among the LACV strains.
(TIF)

S2 Fig Multiple sequence alignment of the G2 proteins sequences among the LACV strains.
(TIF)

S3 Fig The phylogenetic tree of G1, G2 and N proteins retrieved from the LACV.
(TIF)

S4 Fig The secondary structure of the LACV-mVax01 predicted by PSIPRED (A), GOR4 (B) and SOPMA (C).
The first bar (Conf) represents the level of confidence in the prediction, with the length of the bar demonstrating varying levels of confidence. The second bar (Cart) utilizes color coding to illustrate the vaccine’s unique structural components. The beta-sheet is symbolized by the color yellow, the helix by the color pink, and the coil structure by the gray coloration. The third bar, “Pred,” and the fourth bar, “AA,” denote distinctive amino acid sequences and structural attributes, respectively.
(TIF)

S5 Fig The post-simulation analyses including RMSD (A), RMSF (B), SASA (C) and ROG (D) of the LACV-mVax01_apo complex.
(TIF)

S6 Fig The H-bond analysis of the LACV-mVax01_TLR2 (A) and LACV-mVax01_TLR4 (B) complexes.
(TIF)

S1 Table List of chosen MHC-I epitopes from G1, G2, and N protein sequences, together with their antigenicity, allergenicity, and toxicity.
(DOCX)

S2 Table List of chosen MHC-II epitopes from G1, G2 and N proteins with their antigenicity, allergenicity, IFN-γ and IL-10 inducing capability, and toxicity.
(DOCX)

S3 Table List of selected linear B-lymphocyte epitopes with their toxicity, allergenicity and antigenicity.
(DOCX)

S4 Table The characteristics of the LACV-mVax01’s secondary structure are assessed by GOR4 and SOPMA.
(DOCX)

S5 Table Discotope 2.0 predicted the conformational B-lymphocyte epitopes residues of the LACV-mVax01.
(DOCX)

S6 Table Post-simulation analyses (RMSD, RMSF, SASA and ROG of the LACV-mVax01 and its complexes.
(DOCX)
