# Supplementary figures and images for: System immunoinformatics–based design of a multi-epitope vaccine candidate against La Crosse virus
Source: PLoS One. 2026 May 28;21(5):e0350287. doi: 10.1371/journal.pone.0350287 (PMC13218471; doi:10.1371/journal.pone.0350287)

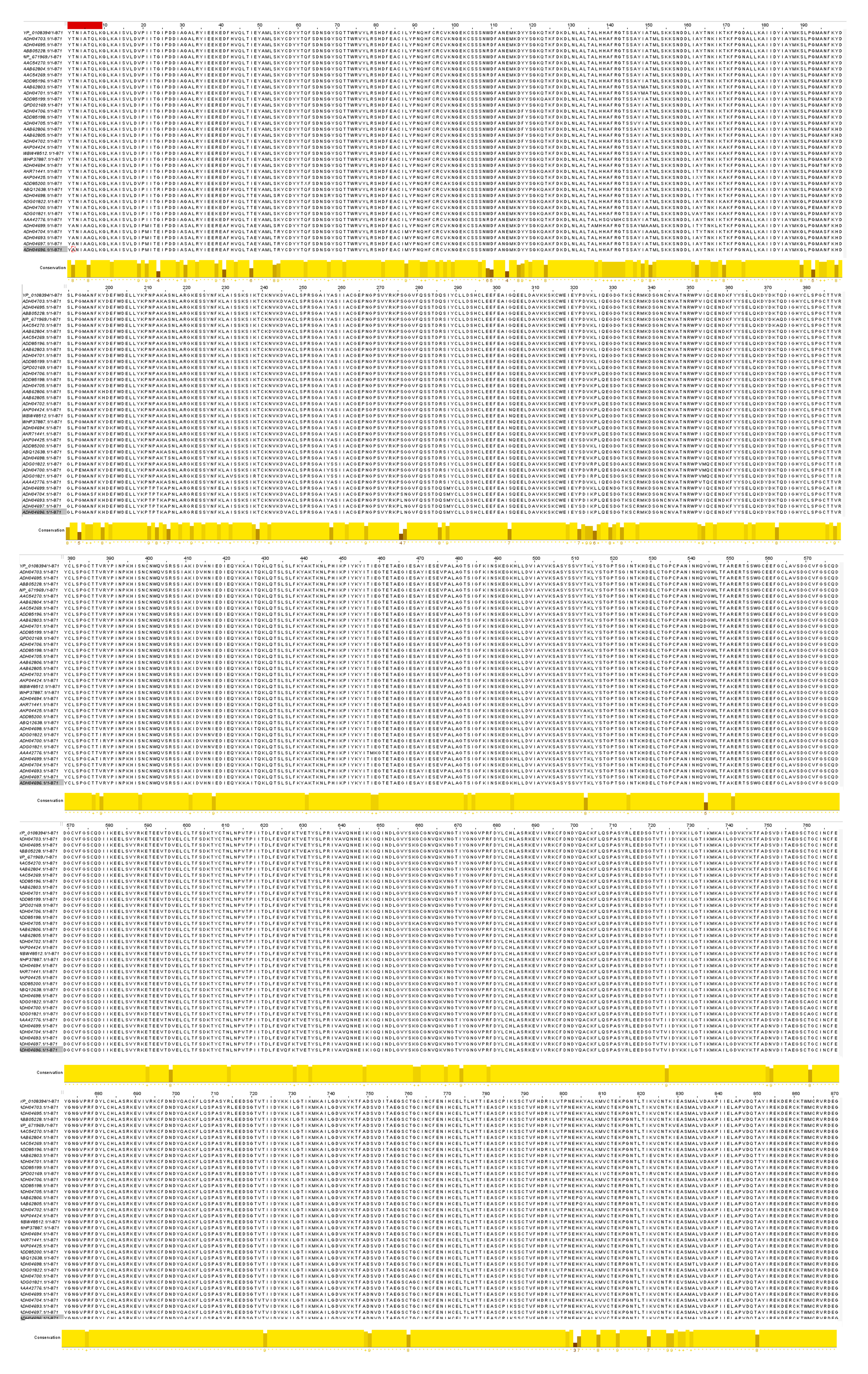

Supplement: S1 Fig — Multiple sequence alignment of the G1 proteins sequences among the LACV strains. (TIF) [file pone.0350287.s001.tif]

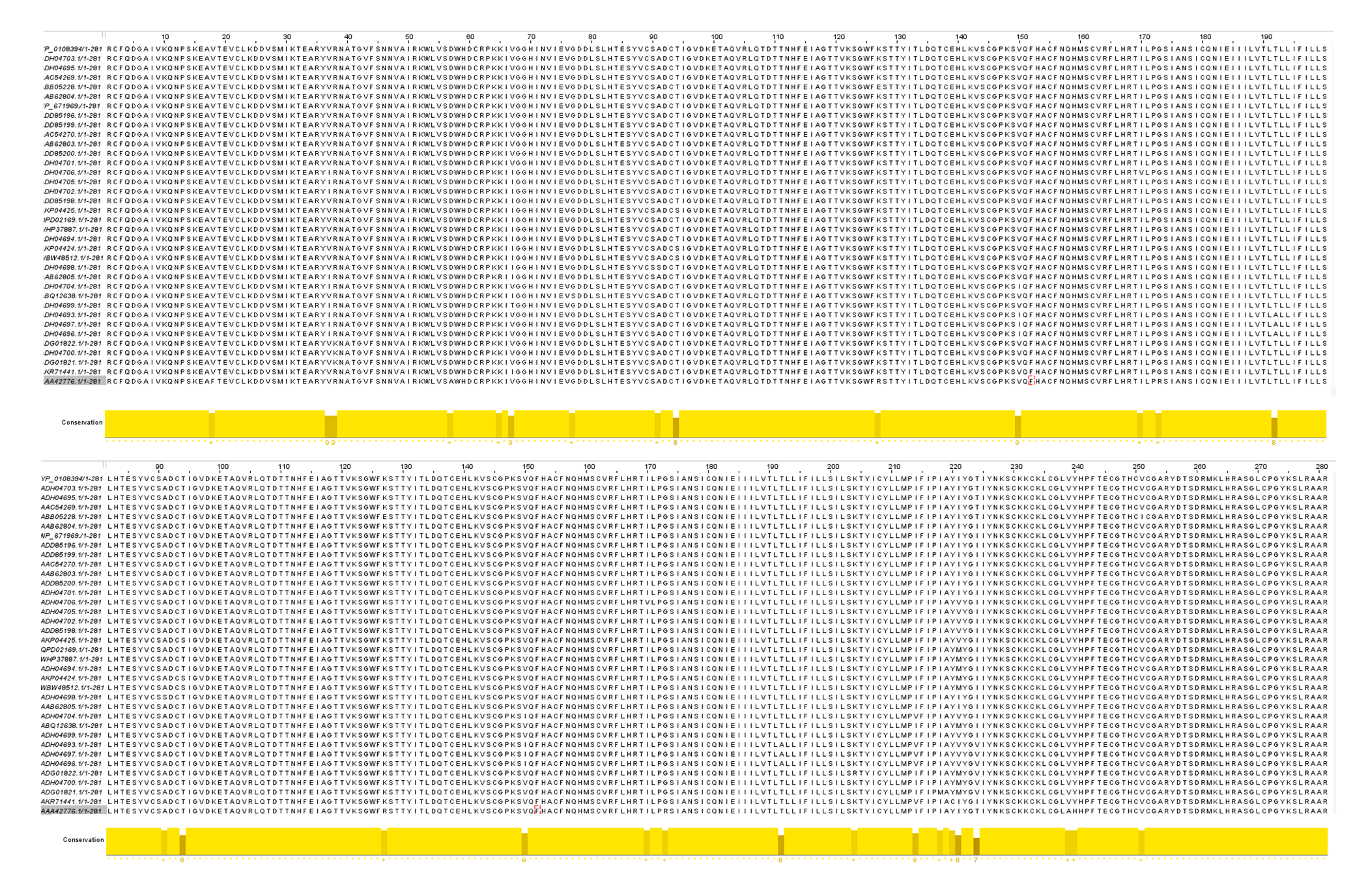

Supplement: S2 Fig — Multiple sequence alignment of the G2 proteins sequences among the LACV strains. (TIF) [file pone.0350287.s002.tif]

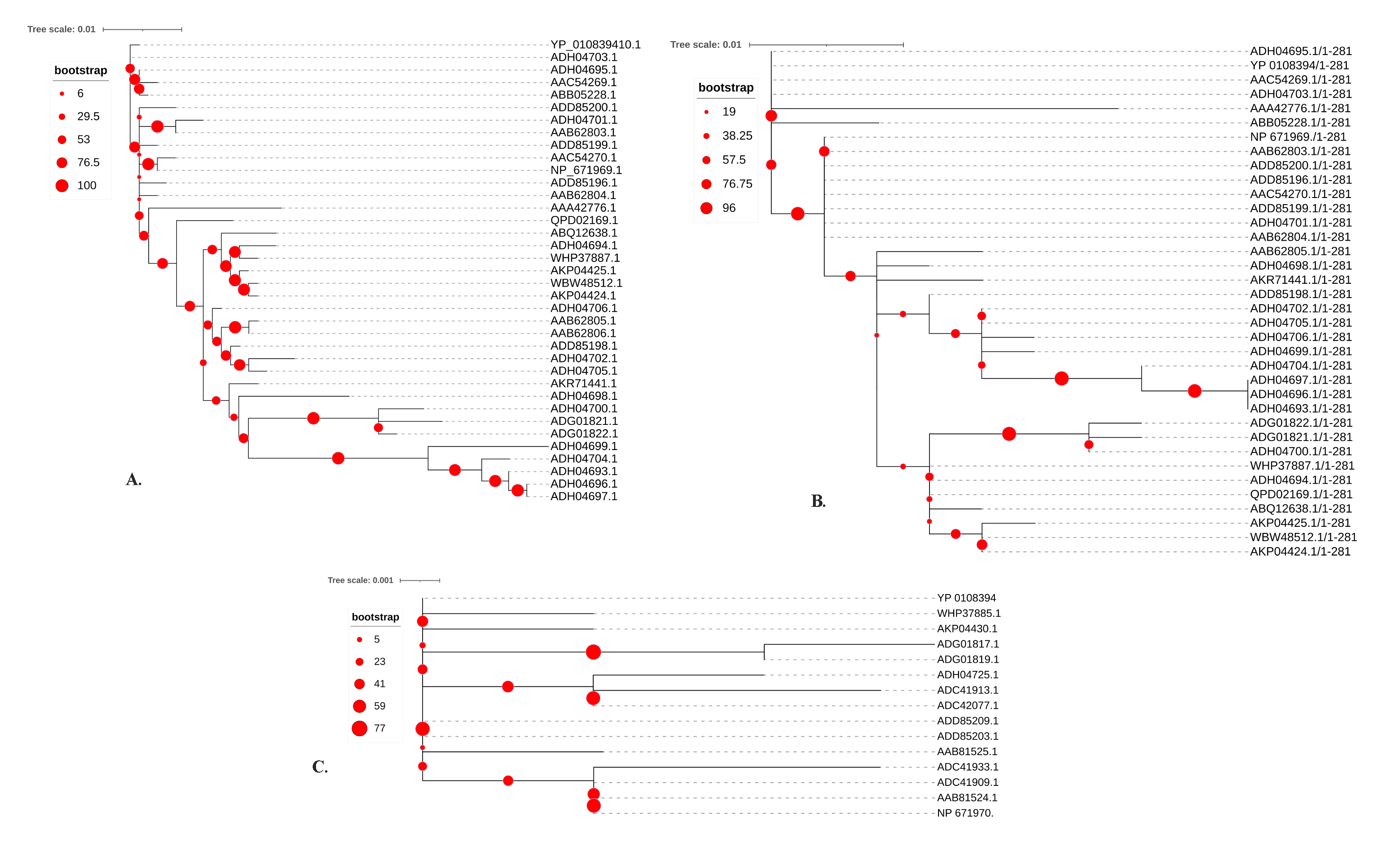

Supplement: S3 Fig — The phylogenetic tree of G1, G2 and N proteins retrieved from the LACV. (TIF) [file pone.0350287.s003.tif]

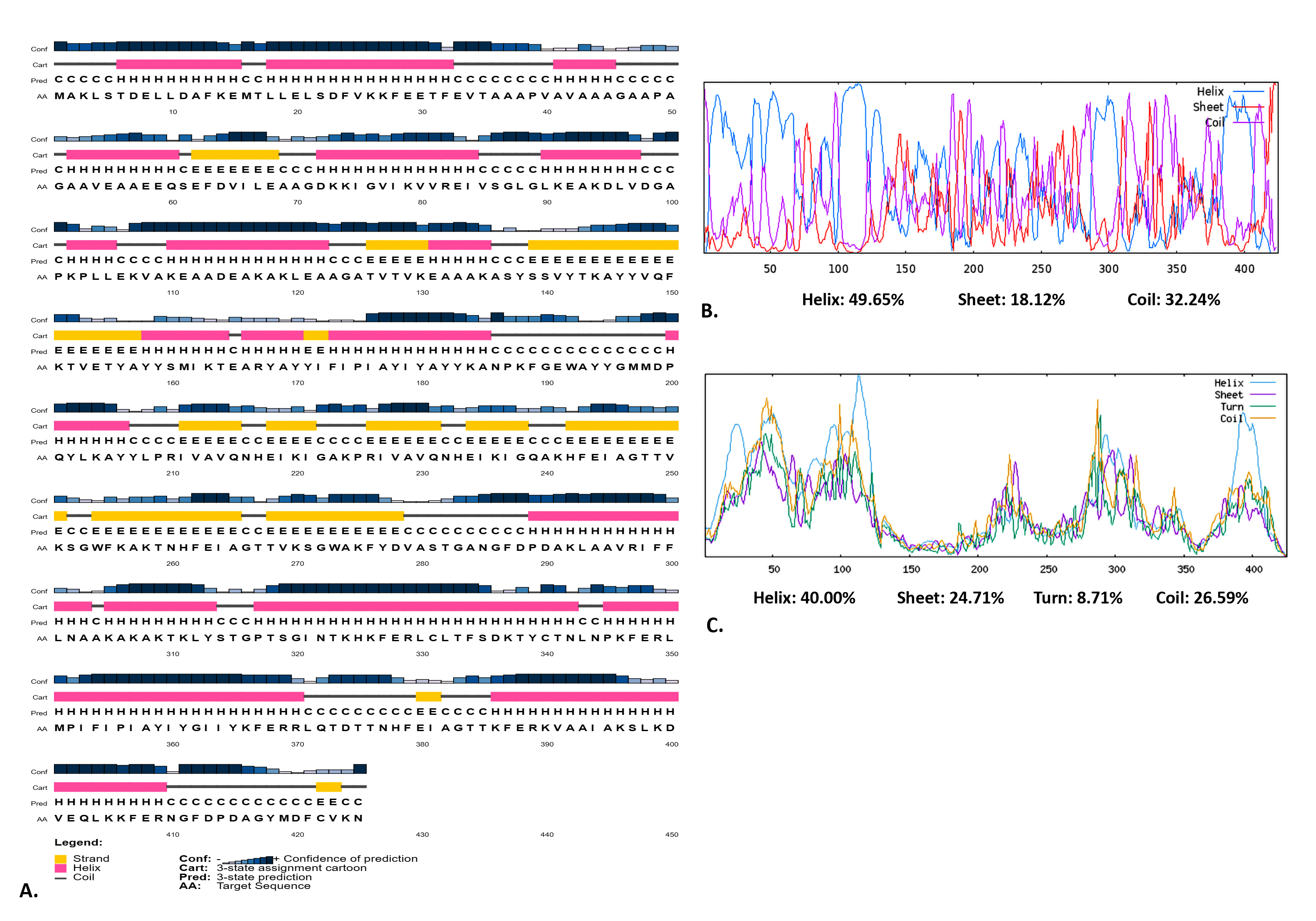

Supplement: S4 Fig — The secondary structure of the LACV-mVax01 predicted by PSIPRED (A), GOR4 (B) and SOPMA (C). The first bar (Conf) represents the level of confidence in the prediction, with the length of the bar demonstrating varying levels of confidence. The second bar (Cart) utilizes color coding to illustrate the vaccine’s unique structural components. The beta-sheet is symbolized by the color yellow, the helix by the color pink, and the coil structure by the gray coloration. The third bar, “Pred,” and the fourth bar, “AA,” denote distinctive amino acid sequences and structural attributes, respectively. (TIF) [file pone.0350287.s004.tif]

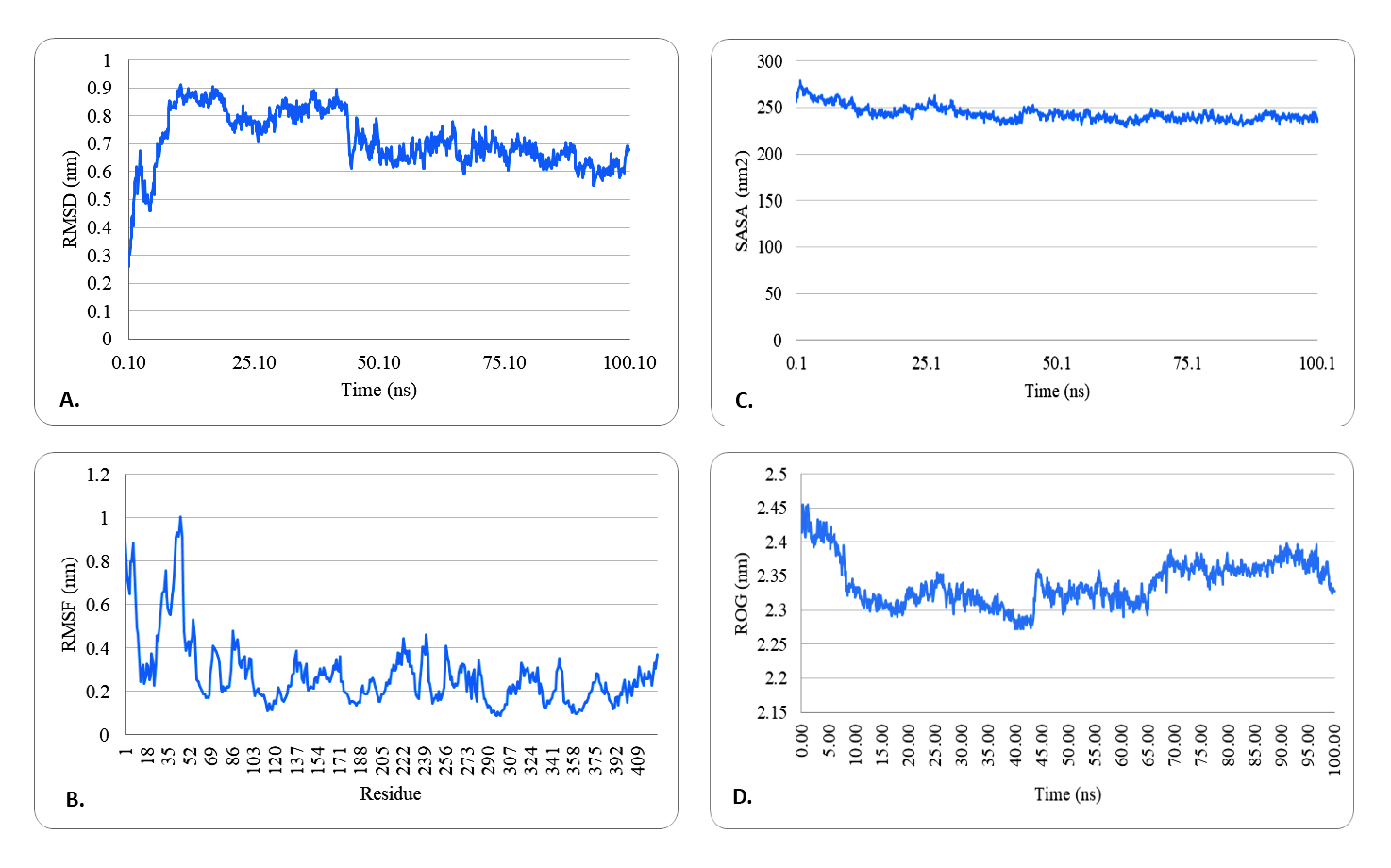

Supplement: S5 Fig — The post-simulation analyses including RMSD (A), RMSF (B), SASA (C) and ROG (D) of the LACV-mVax01_apo complex. (TIF) [file pone.0350287.s005.tif]

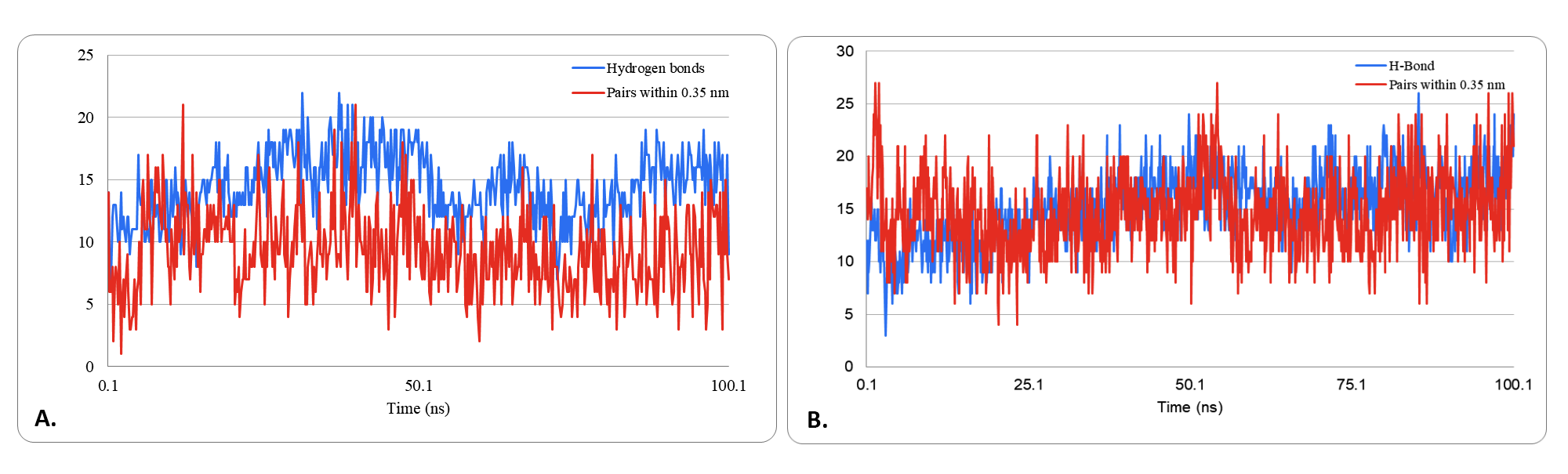

Supplement: S6 Fig — The H-bond analysis of the LACV-mVax01_TLR2 (A) and LACV-mVax01_TLR4 (B) complexes. (TIF) [file pone.0350287.s006.tif]
