# Supplementary material for: System immunoinformatics–based design of a multi-epitope vaccine candidate against La Crosse virus
Source: PLoS One. 2026 May 28;21(5):e0350287. doi: 10.1371/journal.pone.0350287 (PMC13218471; doi:10.1371/journal.pone.0350287)
Supplement: S1 Table — List of chosen MHC-I epitopes from G1, G2, and N protein sequences, together with their antigenicity, allergenicity, and toxicity. (DOCX) [file pone.0350287.s007.docx]

**Table S1.** List of chosen MHC-I epitopes from G1, G2, and N protein sequences, together with their antigenicity, allergenicity, and toxicity.

| Protein | Peptide | Percentile rank | Allele | Antigenicity | Allergenicity | Toxicity | Proteosome score | TAP score | Processing score |
| --- | --- | --- | --- | --- | --- | --- | --- | --- | --- |
| G1 | ASYSSVYTK | 0.01 | 27 | Yes | No | No | 0.83 | 0.39 | 1.22 |
|  | VQFKTVETY | 0.01 | 27 | Yes | No | No | 1.50 | 1.31 | 2.80 |
| G2 | SMIKTEARY | 0.02 | 27 | Yes | No | No | 1.20 | 1.40 | 2.59 |
|  | IFIPIAYIY | 0.05 | 27 | Yes | No | No | 1.61 | 1.32 | 2.93 |
| N | KANPKFGEW | 0.02 | 27 | Yes | No | No | 1.28 | 0.47 | 1.75 |
|  | GMMDPQYLK | 0.02 | 27 | Yes | No | No | 1.02 | 0.18 | 1.21 |
