# Supplementary material for: System immunoinformatics–based design of a multi-epitope vaccine candidate against La Crosse virus
Source: PLoS One. 2026 May 28;21(5):e0350287. doi: 10.1371/journal.pone.0350287 (PMC13218471; doi:10.1371/journal.pone.0350287)
Supplement: S2 Table — List of chosen MHC-II epitopes from G1, G2 and N proteins with their antigenicity, allergenicity, IFN-γ and IL-10 inducing capability, and toxicity. (DOCX) [file pone.0350287.s008.docx]

**Table S2**. List of chosen MHC-II epitopes from G1, G2 and N proteins with their antigenicity, allergenicity, IFN-γ and IL-10 inducing capability, and toxicity.

| Protein | Peptide | Adjusted rank | Allele | Antigenicity | Allergenicity | IFN gamma | IL10 inducer | Toxicity |
| --- | --- | --- | --- | --- | --- | --- | --- | --- |
| G1 | LPRIVAVQNHEIKIG | 0.03 | 27 | Yes | No | Positive | No | No |
|  | PRIVAVQNHEIKIGQ | 0.04 | 27 | Yes | No | Positive | No | No |
| G2 | HFEIAGTTVKSGWFK | 0.07 | 27 | Yes | No | Positive | No | No |
|  | TNHFEIAGTTVKSGW | 0.14 | 27 | Yes | No | Positive | No | No |
| N | FYDVASTGANGFDPD | 0.37 | 27 | Yes | No | Positive | No | No |
|  | LAAVRIFFLNAAKAK | 0.51 | 27 | Yes | No | Positive | No | No |
