# Supplementary material for: System immunoinformatics–based design of a multi-epitope vaccine candidate against La Crosse virus
Source: PLoS One. 2026 May 28;21(5):e0350287. doi: 10.1371/journal.pone.0350287 (PMC13218471; doi:10.1371/journal.pone.0350287)
Supplement: S3 Table — List of selected linear B-lymphocyte epitopes with their toxicity, allergenicity and antigenicity. (DOCX) [file pone.0350287.s009.docx]

**Table S3.** List of selected linear B-lymphocyte epitopes with their toxicity, allergenicity and antigenicity.

| Protein | Start | Peptides | Antigenicity | Allergenicity | Toxicity |
| --- | --- | --- | --- | --- | --- |
| G1 | 513 | TKLYSTGPTSGINTKH | Yes | Yes | Yes |
|  | 599 | LCLTFSDKTYCTNLNP | Yes | Yes | Yes |
| G2 | 211 | LMPIFIPIAYIYGIIY | Yes | Yes | Yes |
|  | 105 | RLQTDTTNHFEIAGTT | Yes | Yes | Yes |
| N | 198 | KVAAIAKSLKDVEQLK | Yes | Yes | Yes |
|  | 15 | NGFDPDAGYMDFCVKN | Yes | Yes | Yes |
