# Supplementary material for: System immunoinformatics–based design of a multi-epitope vaccine candidate against La Crosse virus
Source: PLoS One. 2026 May 28;21(5):e0350287. doi: 10.1371/journal.pone.0350287 (PMC13218471; doi:10.1371/journal.pone.0350287)
Supplement: S4 Table — The characteristics of the LACV-mVax01’s secondary structure are assessed by GOR4 and SOPMA. (DOCX) [file pone.0350287.s010.docx]

**Table S4.** The characteristics of the LACV-mVax01’s secondary structure are assessed by GOR4 and SOPMA.

| Properties | GOR4 | SOPMA |
| --- | --- | --- |
| Alpha helix | 211 (49.65%) | 170 (40.00%) |
| 3_10_ helix | 0.00% | 0.00% |
| Pi helix | 0.00% | 0.00% |
| Beta bridge | 0.00% | 0.00% |
| Extended strand | 77 (18.12%) | 105 (24.71%) |
| Beta turn | 0.00% | 37 (8.71%) |
| Bend region | 0.00% | 0.00% |
| Random coil | 137 (32.24%) | 113 (26.59%) |
