# Supplementary material for: System immunoinformatics–based design of a multi-epitope vaccine candidate against La Crosse virus
Source: PLoS One. 2026 May 28;21(5):e0350287. doi: 10.1371/journal.pone.0350287 (PMC13218471; doi:10.1371/journal.pone.0350287)
Supplement: S5 Table — Discotope 2.0 predicted the conformational B-lymphocyte epitopes residues of the LACV-mVax01. (DOCX) [file pone.0350287.s011.docx]

**Table S5.** Discotope 2.0 predicted the conformational B-lymphocyte epitopes residues of the LACV-mVax01.

| No | Residues | Number of residues | Scores |
| --- | --- | --- | --- |
| 1 | A:Y339, A:C340, A:T341, A:N342, A:L343, A:N344, A:P345, A:K346 | 8 | 0.843 |
| 2 | A:M1, A:A2, A:K3, A:L4, A:S5, A:T6, A:D7, A:E8, A:L9, A:L10, A:D11, A:A12, A:F13, A:K14, A:E15, A:M16, A:T17, A:L18, A:L19, A:E20, A:L21, A:S22, A:D23, A:F24, A:V25, A:K26, A:K27, A:F28, A:E29, A:E30, A:T31, A:F32, A:E33, A:V34, A:T35, A:A36, A:A37, A:A38, A:P39, A:V40, A:A41, A:V42, A:A43, A:A44, A:A45, A:G46, A:A47, A:A48, A:P49, A:A50, A:G51, A:A52, A:A53, A:V54, A:E55, A:A56 | 56 | 0.835 |
| 3 | A:A303, A:Y313, A:S314, A:T315, A:G316, A:P317, A:T318, A:S319, A:G320, A:I321, A:N322, A:T323, A:K324, A:H325, A:K326, A:R329, A:F347, A:E348, A:R349, A:L350, A:M351, A:P352, A:I353, A:K399, A:D400, A:E402, A:Q403, A:L404, A:K405, A:K406, A:F407, A:E408, A:R409, A:N410, A:G411, A:F412, A:D413, A:P414, A:D415, A:A416, A:G417, A:Y418, A:M419, A:D420, A:F421, A:C422, A:V423, A:K424, A:N425 | 49 | 0.727 |
| 4 | A:K311, A:L371, A:Q372, A:T373, A:D374, A:T375, A:T376, A:N377, A:H378 | 9 | 0.696 |
| 5 | A:Y156, A:A157, A:Y159, A:S160, A:M161, A:I162, A:K163, A:T164, A:E165, A:A166, A:R167, A:Y168, A:A169, A:Y170, A:Y171, A:F173, A:I264 | 17 | 0.67 |
| 6 | A:H233, A:E234, A:I235, A:K236, A:I237, A:G238, A:Q239, A:A240, A:K241, A:H242, A:F243, A:E244, A:I245, A:G247, A:F379, A:I381, A:A382, A:G383, A:T384, A:T385, A:K386, A:F387, A:E388, A:R389, A:K390 | 25 | 0.636 |
| 7 | A:E68, A:A69, A:A70, A:G71, A:D72, A:K73, A:K74, A:I75, A:G76, A:I78, A:K79, A:R82, A:E83, A:I84, A:V85, A:S86, A:G87, A:L88, A:G89, A:L90, A:K91, A:E92, A:A93, A:K94, A:D95, A:L96, A:V97, A:D98, A:G99, A:A100, A:P101, A:K102, A:G124, A:A125, A:K251, A:S252 | 36 | 0.588 |
| 8 | A:S335, A:D336, A:K337, A:T338 | 4 | 0.574 |
| 9 | A:E131, A:A132, A:A133, A:A134, A:K135, A:A136, A:S137, A:Y138, A:S139, A:S140, A:V141, A:W192, A:W254, A:K256, A:A257, A:K258, A:T259, A:N260, A:H261, A:F262, A:E263 | 21 | 0.563 |
| 10 | A:V148, A:Q149, A:F150 | 3 | 0.537 |
| 11 | A:G272, A:A274, A:K275, A:D278 | 4 | 0.508 |
