# Supplementary material for: System immunoinformatics–based design of a multi-epitope vaccine candidate against La Crosse virus
Source: PLoS One. 2026 May 28;21(5):e0350287. doi: 10.1371/journal.pone.0350287 (PMC13218471; doi:10.1371/journal.pone.0350287)
Supplement: S6 Table — Post-simulation analyses (RMSD, RMSF, SASA and ROG of the LACV-mVax01 and its complexes. (DOCX) [file pone.0350287.s012.docx]

**Table S6.** Post-simulation analyses (RMSD, RMSF, SASA and ROG of the LACV-mVax01 and its complexes

|  | LACV-mVax01_apo | LACV-mVax01_TLR2 | TLR2 | LACV-mVax01 | LACV-mVax01_TLR4 | TLR4 | LACV-mVax01 |
| --- | --- | --- | --- | --- | --- | --- | --- |
| RMSD (nm) | 0.71 | 0.66 | 0.26 | 0.68 | 0.56 | 0.19 | 0.75 |
| RMSF (nm) | 0.28 | 1.36 | 1.33 | 1.40 | 0.16 | 0.12 | 0.24 |
| SASA (nm^2^) | 243.30 | 471.81 | 253.46 | 244.52 | 472.22 | 267.89 | 247.21 |
| ROG (nm) | 2.34 | 3.89 | 2.98 | 2.39 | 3.28 | 3.21 | 2.39 |
